# Supplementary material for: A titin missense variant drives atrial electrical remodeling and is associated with atrial fibrillation
Source: medRxiv. 2025 Nov 7:2024.12.06.24318402. Originally published 2024 Dec 8. Preprint. [Version 3] doi: 10.1101/2024.12.06.24318402 (PMC11643245; doi:10.1101/2024.12.06.24318402)
Supplement: Supplement 1 [file NIHPP2024.12.06.24318402v3-supplement-1.pdf]

medRxiv preprint doi: https://doi.org/10.1101/2024.12.06.24318402; this version posted November 7, 2025. The copyright holder for this preprint (which was not certified by peer review) is the author/funder, who has granted medRxiv a license to display the preprint in perpetuity.
It is made available under a CC-BY 4.0 International license .

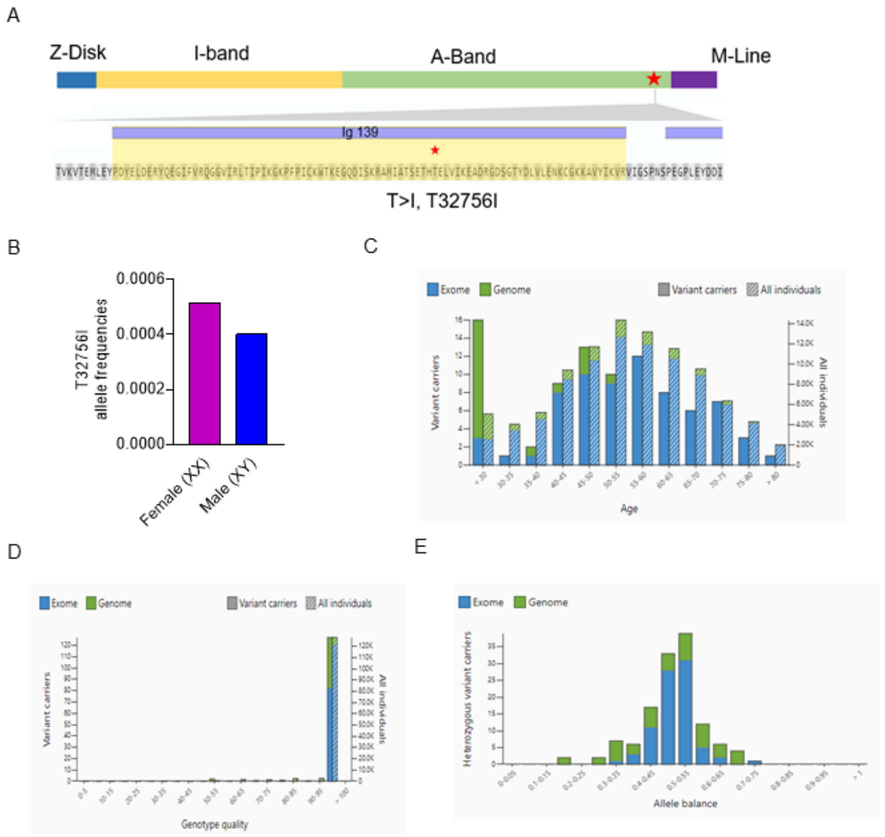

Supplementary Figure 1: *TTN*-T32756I position and distribution. (A) Location of the T23756I at Ig139 domain in the A-band of titin. (B) Allele frequencies of T3265I between sexes. (C) Age distribution of the variant carriers. (D) Genotype quality. (E) Allele balance for heterozygotes. Source: https://gnomad.broadinstitute.org/variant/2-179404525-G-A?dataset=gnomad_r2_1.

medRxiv preprint doi: https://doi.org/10.1101/2024.12.06.24318402; this version posted November 7, 2025. The copyright holder for this preprint (which was not certified by peer review) is the author/funder, who has granted medRxiv a license to display the preprint in perpetuity.
It is made available under a CC-BY 4.0 International license .

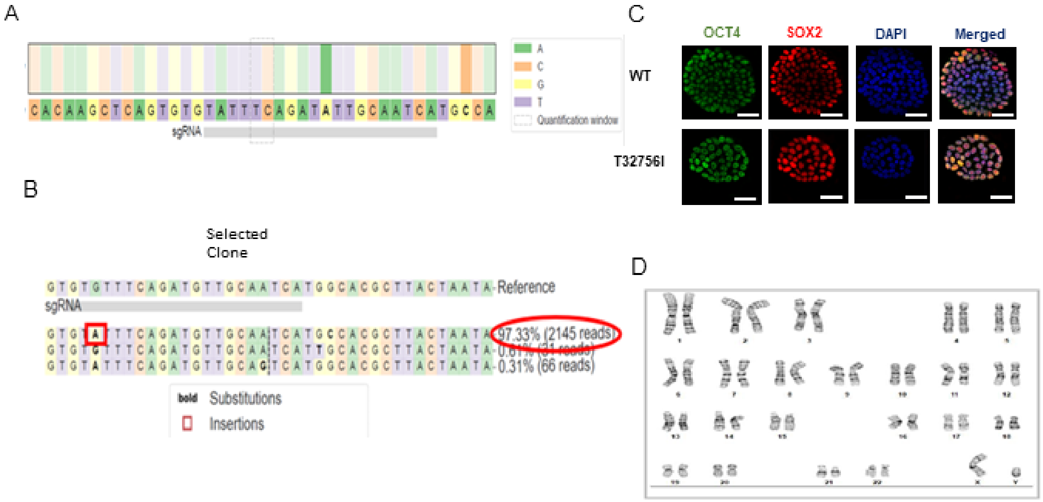

Supplementary Figure 2: Generation of iPSC-aCMs with *TTN*-T32756I. (A) *TTN* gene locus for the generation of isogenic iPSCs with the T32756I variation. Guide sequence (gRNA) shown in the bottom gray box was cloned into the vector to express gRNA guiding Cas9 exonuclease to the targeted protospacer adjacent motif sequence. (B) Next-generation sequencing of the confirming T32756I mutation. (C) Representative immunostaining of pluripotency markers OCT4 and SOX2 in iPSCs. The 4′,6-diamidino-2-phenylindole (DAPI) indicates the nucleus. (D) Karyotype analysis of the T32756I iPSCs.

medRxiv preprint doi: https://doi.org/10.1101/2024.12.06.24318402; this version posted November 7, 2025. The copyright holder for this preprint (which was not certified by peer review) is the author/funder, who has granted medRxiv a license to display the preprint in perpetuity.
It is made available under a CC-BY 4.0 International license .

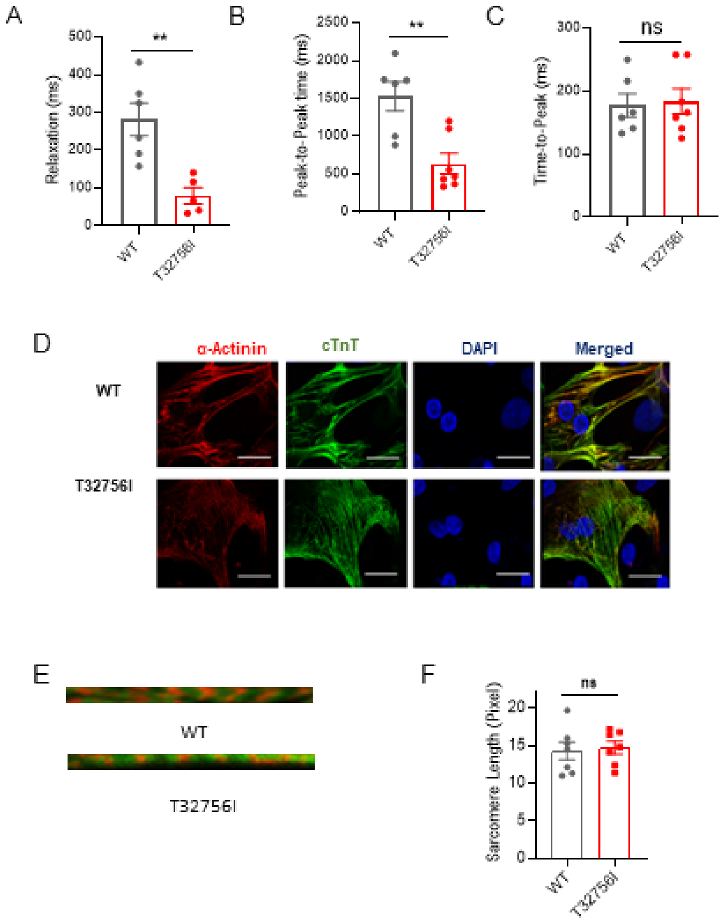

Supplementary Figure 3: Contractility and sarcomere organization of *TTN*-T32756I iPSC-aCMs. (A-C) Compared to WT, *TTN*-T32756I (Red) iPSC-aCMs show decreased relaxation time (A) and peak-to-peak time (B), but no significant change to time-to-peak (C). (D-E) Immunostaining showing the sarcomeric organization of WT and *TTN*-T32756I iPSC-aCM by the pan-cardiomyocyte (CM) marker cardiac troponin T (cTnT; green) and α-actinin (orange). The DAPI staining indicates the nucleus. (F) Bar graph showing no change in the sarcomere length. n.s. P>0.05; **P<0.01.

medRxiv preprint doi: https://doi.org/10.1101/2024.12.06.24318402; this version posted November 7, 2025. The copyright holder for this preprint (which was not certified by peer review) is the author/funder, who has granted medRxiv a license to display the preprint in perpetuity.
It is made available under a CC-BY 4.0 International license .

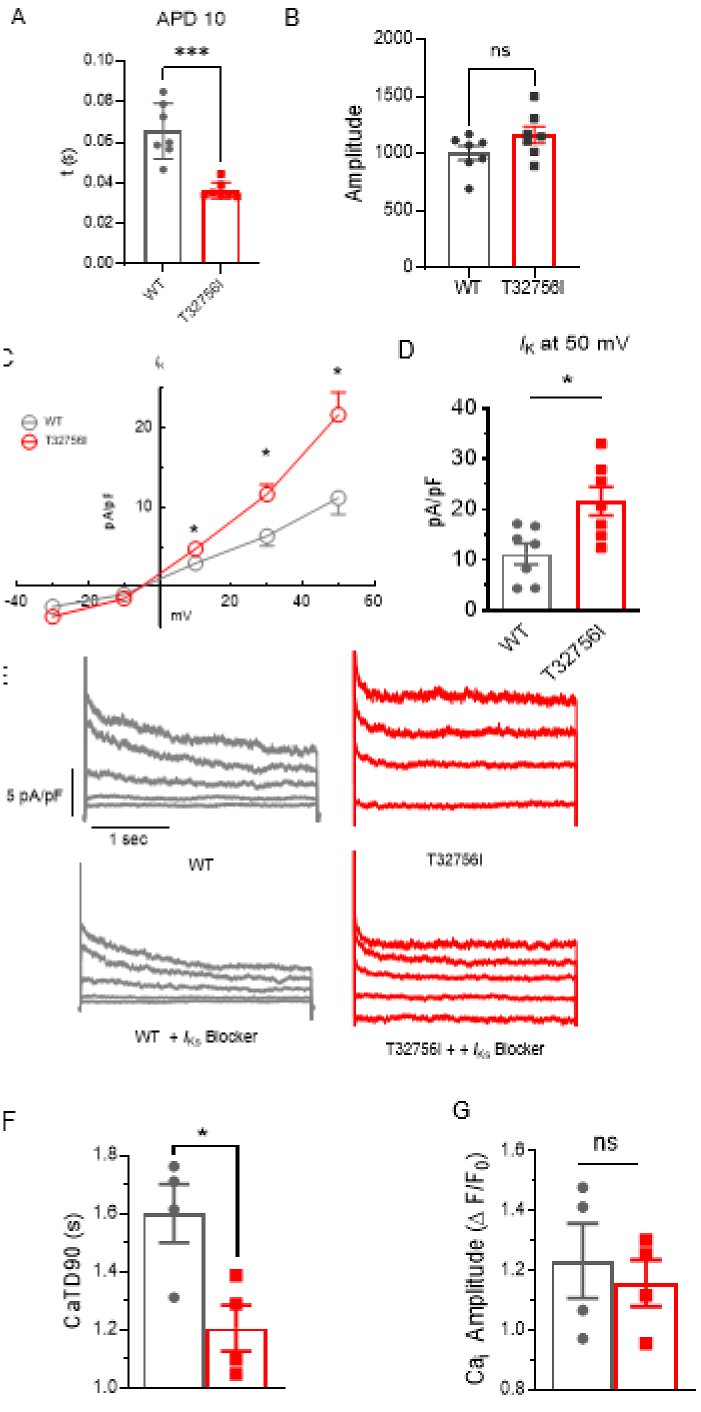

Supplementary Figure 4: *TTN*-T32756I iPSC-aCMs display anomalous action potentials, potassium currents, and calcium-handling. (A) Compared to the WT, *TTN*-T32756I shows reduction of action potential duration at the 10% (APD10) repolarization. (B) Bar graph showing no change in the amplitude of the AP. (C) Total

medRxiv preprint doi: https://doi.org/10.1101/2024.12.06.24318402; this version posted November 7, 2025. The copyright holder for this preprint (which was not certified by peer review) is the author/funder, who has granted medRxiv a license to display the preprint in perpetuity.
It is made available under a CC-BY 4.0 International license .

potassium current (I_K) and voltage relationship (I-V curves) in WT and *TTN*-T32756I iPSC-aCMs. (D) Total I_K current density at 50 mV. (E) Representative current traces at different voltages showing the isolation of the I_{ks} current with the selective blocker HMR-1556 in both WT and *TTN*-T32756I iPSC-aCMs. (F-G) Bar graph showing that the *TTN*-T32756I iPSC-aCMs have decreased transient durations (F), but no change in the transient peak amplitudes (G) compared with the WT iPSC-aCMs. n.s. P>0.05; *P<0.05; ***P<0.001.

medRxiv preprint doi: https://doi.org/10.1101/2024.12.06.24318402; this version posted November 7, 2025. The copyright holder for this preprint (which was not certified by peer review) is the author/funder, who has granted medRxiv a license to display the preprint in perpetuity.
It is made available under a CC-BY 4.0 International license .

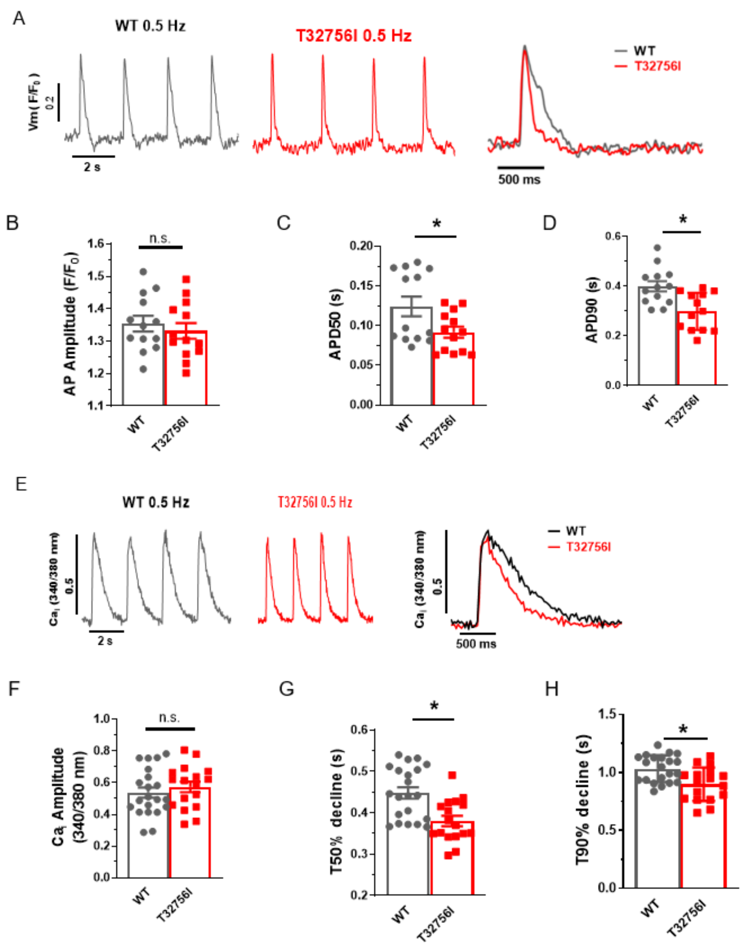

Supplementary Figure 5: Effect of T32756I on action potential and calcium-handling in paced iPSC-aCMs. (A) Representative optical AP recordings of paced (0.5 Hz) WT and *TTN*-T32756I showing reduction of AP duration (APD). (B) Bar graph showing no change in the amplitude of the AP. (C) APD50 (D) APD90. (D) Representative tracings of paced calcium transients (0.5 Hz) of WT and *TTN*-T32756I iPSC-aCMs. (E) No change in the transient peak amplitudes. (F-G). Decreased transient durations (I) in *TTN*-T32756I iPSC-aCMs compared with the WT iPSC-aCMs. n.s. $P>0.05$; *$P<0.05$.

medRxiv preprint doi: https://doi.org/10.1101/2024.12.06.24318402; this version posted November 7, 2025. The copyright holder for this preprint (which was not certified by peer review) is the author/funder, who has granted medRxiv a license to display the preprint in perpetuity.
It is made available under a CC-BY 4.0 International license .

(A) Compared to the WT, *TTN*-T32756I shows reduction of action potential duration at the 10% (APD10) repolarization. (B) Bar graph showing no change in the amplitude of the AP. (C) Total potassium current (I_K) and voltage relationship (I-V curves) in WT and *TTN*-T32756I iPSC-aCMs. (D) Total I_K current density at 50 mV. (E) Representative current traces at different voltages showing the isolation of the I_{ks} current with the selective blocker HMR-1556 in both WT and *TTN*-T32756I iPSC-aCMs. (F-G) Bar graph showing that the *TTN*-T32756I iPSC-aCMs have decreased transient durations (F), but no change in the transient peak amplitudes (G) compared with the WT iPSC-aCMs. n.s. $P>0.05$; *$P<0.05$.

medRxiv preprint doi: https://doi.org/10.1101/2024.12.06.24318402; this version posted November 7, 2025. The copyright holder for this preprint (which was not certified by peer review) is the author/funder, who has granted medRxiv a license to display the preprint in perpetuity.
It is made available under a CC-BY 4.0 International license .

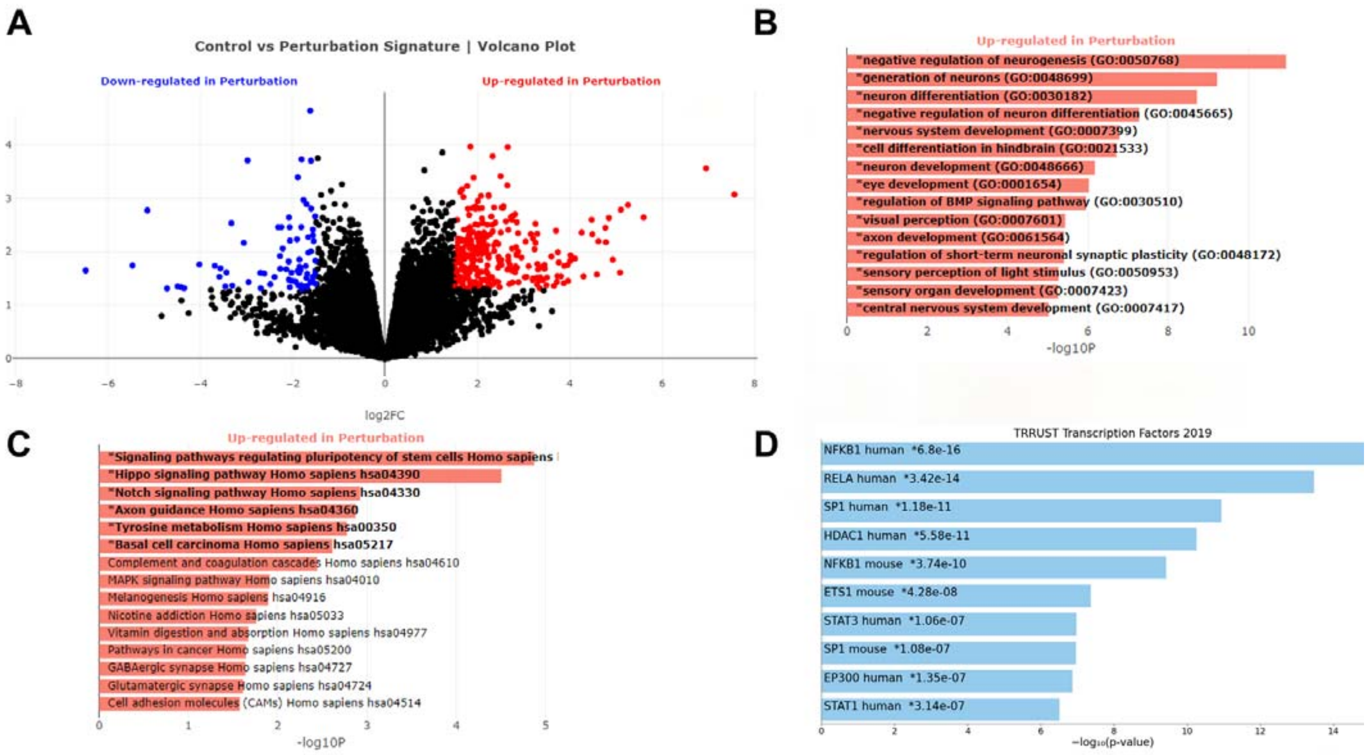

Supplementary Figure 6: Upregulated pathways in *TTN*-T32756I iPSC-aCMs with the WT. (A) Volcano plot showing spread of downregulated and upregulated differentially expressed genes (DEGs) (B) Top significantly enriched upregulated Gene-Ontology Biological process (GO-BP) pathways in the *TTN*-T32756I iPSC-aCMs. (C) Top significantly enriched upregulated Kyoto Encyclopedia of Genes and Genomes (KEGG) pathways in the *TTN*-T32756I iPSC-aCMs. (D) Top significantly enriched TTRUST transcription factors (TFs).

medRxiv preprint doi: https://doi.org/10.1101/2024.12.06.24318402; this version posted November 7, 2025. The copyright holder for this preprint (which was not certified by peer review) is the author/funder, who has granted medRxiv a license to display the preprint in perpetuity.
It is made available under a CC-BY 4.0 International license .

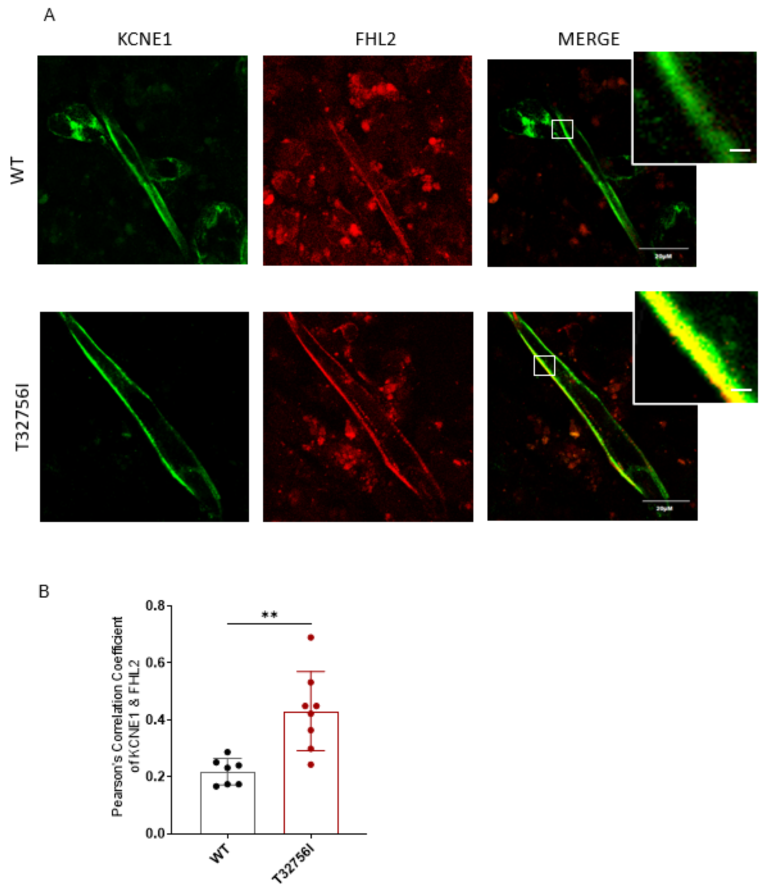

Supplementary Figure 7: Increased FHL2-KCNE1 co-localization in TTN-T32756I iPSC-aCMs. (A) Representative confocal immunofluorescence images showing subcellular localization of FHL2 (red) and KCNE1 (green) in WT and *TTN*-T32756I iPSC-aCMs. (B) Quantitative co-localization analysis, assessed by Pearson's correlation coefficient, demonstrated a significant increase in FHL2–KCNE1 co-localization in *TTN*-T32756I iPSC-aCMs compared with WT. **P < 0.01.

Subject ID	Age	Sex	Race-Ethnicity	Nucleotide	Amino Acid Change	dbSNP	gnomAD Allele Frequency	Exon	Band	Percent Spliced In (PSI)	REVEL Score
1	55-59	F	NHB	c.70250T>C	p.Ile23417Thr	rs201836227	0.000221	326	A-band	100	0.66297
2	50-54	M	HL	c.52022G>A	p.Arg17341Gln	rs370390570	0.000116	273	A-band	100	0.60691
3	50-54	M	HL	c.62519G>A	p.Gly20840Asp	rs1326564200	0.000012	304	A-band	100	0.883
4	55-59	M	NHB	c.59248G>A	p.Gly19750Ser	rs200732032	0.000109	300	A-band	100	0.75279
5	55-59	M	NHB	c.58363G>A	p.Gly19455Ser	rs191927501	0.000157	297	A-band	100	0.80842
6	65-69	M	HL	c.57727G>C	p.Ala19243Pro	rs1313667626	0.000004	295	A-band	100	0.81369
7	40-44	F	NHB	c.101665G>A	p.Val33889Ile	rs34924609	0.003099	358	A-band	100	0.17295
8	40-44	F	NHB	c.6959G>A	p.Arg2320His	rs374615369	0.000076	30	I-band	100	0.82403
9	55-59	M	NHB	c.42145G>T	p.Val14049Leu	rs1206523368			I-band	100	0.73517
10	60-64	M	NHB	c.67808C>T	p.Ala22603Val	rs199583938	0.000036	320	A-band	100	0.42028
11	60-64	M	NHB	c.101557A>G	p.Lys33853Glu	rs727505163	0.000004	358	A-band	100	0.75074
12	75-79	F	NHB	c.93266G>A	p.Arg31089His	rs367993101	0.000028	339	A-band	100	0.87283
13	75-79	F	NHB	c.970C>T	p.Pro324Ser	rs72647845	0.000598	7	Z-disk	100	0.70603
14	55-59	M	HL	c.89426G>A	p.Arg29809Gln	rs72648238	0.000632	334	A-band	100	0.45592
15	55-59	M	HL	c.99433C>T	p.Arg33145Trp	rs1338284042	0.000004	355	A-band	100	0.61955
16	75-79	M	NHB	c.74870A>G	p.Lys24957Arg	rs760043791	0.000004	326	A-band	100	0.43483
17	75-79	M	NHB	c.70817T>C	p.Met23606Thr	rs371030086	0.000040	326	A-band	100	0.624
18	80-84	F	NHB	c.101665G>A	p.Val33889Ile	rs34924609	0.003099	358	A-band	100	0.17295
19	35-39	F	NHB	c.96605T>C	p.Val32202Ala	rs763365622	0.000004	347	A-band	100	0.54781
20	35-39	F	NHB	c.70543T>G	p.Tyr23515Asp				A-band	100	0.67962
21	65-69	M	NHB	c.4671G>A	p.Met1557Ile	rs139192633	0.000272	27	near Z-disk	100	0.26882
22	65-69	F	NHB	c.91937A>G	p.Asn30646Ser	rs72648245	0.000568	338	A-band	100	0.71558
23	65-69	F	NHB	c.46693G>T	p.Ala15565Ser	rs145520397	0.000445	250	I-band	100	0.51417
24	65-69	F	NHB	c.100396C>T	p.Arg33466Cys	rs371908649	0.000145	357	A-band	100	0.8849
25	65-69	F	NHB	c.56693G>A	p.Arg18898His	rs572453785	0.000051	291	A-band	100	0.29647
26	45-49	M	NHB	c.82061T>G	p.Val27354Gly	rs368023868	0.000036	326	A-band	100	0.77847
27	45-49	M	NHB	c.76987G>A	p.Asp25663Asn	rs143186270	0.000105	326	A-band	100	0.49396
28	80-84	F	NHB	c.8938G>A	p.Ala2980Thr	rs72647885	0.000371	38	I-band	100	0.75822
29	70-74	F	NHB	c.9077A>T	p.Asn3026Ile	rs11900987	0.000454	38	I-band	100	0.56024
30	85-89	F	HL	c.7180G>C	p.Glu2394Gln	rs537269762	0.000004	31	I-band	100	0.58254
31	45-49	M	HL	c.57586C>G	p.Leu19196Val	rs397517630	0.000171	295	A-band	100	0.50185
32	60-64	F	NHB	c.44072C>T	p.Thr14691Ile	rs1048028645			I-band	100	0.73445
33	60-64	F	NHB	c.63245C>A	p.Thr21082Asn				A-band	100	0.74377
34	65-69	F	NHB	c.101936C>G	p.Pro33979Arg	rs200238877	0.000213	358	A-band	100	0.73517
35	65-69	F	NHB	c.57683G>A	p.Arg19228His	rs114711705	0.000488	295	A-band	100	0.30369
36	65-69	F	NHB	c.85691A>T	p.Lys28564Ile	rs199859344	0.000443	326	A-band	100	0.69443
37	55-59	M	NHB	c.44525C>T	p.Thr14842Ile	rs370782364	0.000012	241	I-band	100	0.75347
38	55-59	M	NHB	c.81502C>T	p.Arg27168Cys	rs377616334	0.000028	326	A-band	100	0.63269
39	65-69	F	NHB	c.88340C>G	p.Thr29447Arg	rs140201636	0.000198	331	A-band	100	0.58883
40	65-69	F	NHB	c.87137T>G	p.Met29046Arg	rs143975327	0.000192	328	A-band	100	0.81427
41	65-69	F	NHB	c.73316C>T	p.Thr24439Ile	rs750110781	0.000008	326	A-band	100	0.32913
42	60-64	F	NHB	c.98267C>T	p.Thr32756Ile	rs199805060	0.000330	352	A-band	100	0.58758
43	60-64	M	HL	c.91573A>G	p.Ile30525Val	rs72648244	0.006222	337	A-band	100	0.18881
44	60-64	M	HL	c.72931A>G	p.Thr24311Ala	rs56201325	0.003985	326	A-band	100	0.1533

medRxiv preprint doi: https://doi.org/10.1101/2024.12.06.24318402; this version posted November 7, 2025. The copyright holder for this preprint (which was not certified by peer review) is the author/funder, who has granted medRxiv a license to display the preprint in perpetuity.
It is made available under a CC-BY 4.0 International license .

Subject ID	Age	Sex	Race-Ethnicity	Nucleotide	Amino Acid Change	dbSNP	gnomAD Allele Frequency	Exon	Band	Percent Spliced In (PSI)	REVEL Score
45	60-64	F	NHB	c.8938G>A	p.Ala2980Thr	rs72647885	0.000371	38	I-band	100	0.75822
46	65-69	M	NHB	c.79612A>G	p.Thr26538Ala	rs150682764	0.000322	326	A-band	100	0.70029
47	65-69	M	HL	c.14911T>G	p.Cys4971Gly	rs537312655	0.000439	50	I-band	100	0.3358
48	55-59	M	NHB	c.43622C>T	p.Ser14541Leu	rs768180052	0.000008	236	I-band	100	0.60219
49	40-44	F	NHB	c.69883G>A	p.Ala23295Thr	rs746519147	0.000032	326	A-band	100	0.21907
50	75-79	M	NHB	c.52927C>T	p.Arg17643Trp	rs375944265	0.000060	276	A-band	100	0.75279
51	75-79	M	NHB	c.103363C>T	p.Arg34455Cys	rs72629785	0.000716	358	A-band	100	0.75687
52	75-79	M	NHB	c.81539T>C	p.Ile27180Thr	rs182126530	0.000669	326	A-band	100	0.64318
53	75-79	M	NHB	c.44077C>T	p.Arg14693Cys	rs200445568	0.000169	238	I-band	100	0.68844
54	60-64	M	NHB	c.103363C>T	p.Arg34455Cys	rs72629785	0.000716	358	A-band	100	0.75687
55	75-79	M	NHB	c.61481T>C	p.Ile20494Thr	rs374845737	0.000022	304	A-band	100	0.7293
56	50-54	F	NHB	c.54348A>T	p.Glu18116Asp	rs773746281	0.000016	281	A-band	100	0.48268
57	70-74	M	NHB	c.103906C>T	p.Arg34636Cys	rs768575577	0.000028	358	A-band	100	0.59861
58	70-74	M	NHB	c.97892A>T	p.Lys32631Ile	rs944963846	0.000004	351	A-band	100	0.56959
59	50-54	F	NHB	c.2765G>A	p.Arg922His	rs56046320	0.000703	16	near Z-disk	99	0.27141
60	50-54	F	NHB	c.60104G>A	p.Cys20035Tyr	rs774488793			A-band	100	0.71869
61	50-54	F	NHB	c.55079C>T	p.Pro18360Leu	rs192788942	0.000117	283	A-band	100	0.76881
62	90-94	M	HL	c.95414T>G	p.Phe31805Cys				A-band	100	0.73005
63	90-94	M	HL	c.74504A>G	p.Tyr24835Cys	rs201724962	0.000069	326	A-band	100	0.80901
64	55-59	M	NHB	c.78896T>A	p.Val26299Asp	rs73036377	0.000131	326	A-band	100	0.84613
65	45-49	M	NHB	c.101245G>A	p.Val33749Met	rs201554140	0.000538	358	A-band	100	0.72631
66	45-49	M	NHB	c.4199G>C	p.Ser1400Thr	rs138506461	0.000518	24	near Z-disk	100	0.12618
67	45-49	M	NHB	c.105127C>T	p.Arg35043Cys	rs200378865	0.000462	358	A-band	100	0.79765
68	50-54	M	NHB	c.2599A>G	p.Ser867Gly	rs148631577	0.000084	16	near Z-disk	99	0.27398
69	50-54	M	NHB	c.970C>T	p.Pro324Ser	rs72647845	0.000598	7	Z-disk	100	0.70603
70	55-59	F	NHB	c.69130C>T	p.Pro23044Ser	rs55980498	0.003619	324	A-band	100	0.83802
71	55-59	M	HL	c.95557C>A	p.Arg31853Ser				A-band	100	0.47777
72	55-59	M	HL	c.67989A>T	p.Leu22663Phe	rs1485610846	0.000004	320	A-band	100	0.71401
73	45-49	M	HL	c.88394C>T	p.Ser29465Phe	rs146181116	0.002928	331	A-band	100	0.65325
74	45-49	M	HL	c.89314G>A	p.Glu29772Lys	rs200503016	0.000245	334	A-band	100	0.5787
75	60-64	M	NHB	c.47737C>T	p.Leu15913Phe	rs138576504	0.000399	254	A-band	100	0.60456
76	60-64	M	NHB	c.98893G>A	p.Asp32965Asn	rs186405108	0.000044	353	A-band	100	0.68757
77	70-74	M	HL	c.93392T>G	p.Val31131Gly	rs1176407616	0.000012	339	A-band	100	0.47942
78	40-44	F	NHB	c.97760G>A	p.Arg32587His	rs55704830	0.001734	350	A-band	100	0.50341
79	55-59	F	NHB	c.95876T>A	p.Val31959Glu	rs761732372	0.000008	345	A-band	100	0.70924
80	95-99	F	HL	c.83870G>C	p.Arg27957Thr	rs148067743	0.000145	326	A-band	100	0.23913
81	50-54	F	NHB	c.61322A>G	p.Asn20441Ser	rs147580753	0.000260	304	A-band	100	0.34673
82	50-54	F	NHB	c.8938G>A	p.Ala2980Thr	rs72647885	0.000371	38	I-band	100	0.75822
83	70-74	F	HL	c.102030T>G	p.Ser34010Arg	rs1296387134	0.000024	358	A-band	100	0.69273
84	60-64	F	HL	c.102427A>T	p.Met34143Leu	rs371226574	0.000004	358	A-band	100	0.46781
85	60-64	F	HL	c.96928A>C	p.Thr32310Pro	rs542208825	0.000024	348	A-band	100	0.46444
86	60-64	F	HL	c.56315C>T	p.Thr18772Ile	rs370118111	0.000008	289	A-band	100	0.57482
87	60-64	M	NHB	c.9077A>T	p.Asn3026Ile	rs11900987	0.000454	38	I-band	100	0.56024
88	60-64	M	NHB	c.82411G>A	p.Gly27471Ser	rs757130634			A-band	100	0.31778
89	70-74	M	NHB	c.98893G>C	p.Asp32965His	rs186405108	0.000213	353	A-band	100	0.70276

medRxiv preprint doi: https://doi.org/10.1101/2024.12.06.24318402; this version posted November 7, 2025. The copyright holder for this preprint (which was not certified by peer review) is the author/funder, who has granted medRxiv a license to display the preprint in perpetuity.
It is made available under a CC-BY 4.0 International license .

Subject ID	Age	Sex	Race-Ethnicity	Nucleotide	Amino Acid Change	dbSNP	gnomAD Allele Frequency	Exon	Band	Percent Spliced In (PSI)	REVEL Score
90	70-74	M	NHB	c.72137C>T	p.Ala24046Val	rs146767076	0.000363	326	A-band	100	0.20419
91	70-74	M	NHB	c.72782G>A	p.Arg24261Gln	rs142874389	0.000574	326	A-band	100	0.65522
92	70-74	M	NHB	c.55951G>A	p.Glu18651Lys				A-band	100	0.6251
93	70-74	M	NHB	c.44965A>G	p.Ile14989Val	rs755040094	0.000004	244	I-band	100	0.50496
94	70-74	M	NHB	c.88973T>C	p.Ile29658Thr	rs750026544	0.000024	333	A-band	100	0.16972
95	60-64	M	NHB	c.106439A>G	p.His35480Arg	rs766337455			M-band	100	0.25558
96	55-59	F	NHB	c.69383C>A	p.Ser23128Tyr	rs72646882	0.000576	324	A-band	100	0.76554
97	75-79	F	NHB	c.47770T>A	p.Leu15924Met				A-band	100	0.83636
98	25-29	M	HL	c.64997C>T	p.Ala21666Val	rs1396380194	0.000004	311	A-band	100	0.5065
99	25-29	M	HL	c.106349C>G	p.Thr35450Ser	rs371022420	0.000045	358	A-band	100	0.30369
100	25-29	M	HL	c.97760G>C	p.Arg32587Pro	rs55704830	0.000393	350	A-band	100	0.58633
101	25-29	M	HL	c.57165A>T	p.Glu19055Asp	rs1263660973	0.000004	293	A-band	100	0.58507
102	25-29	M	HL	c.73168A>G	p.Thr24390Ala	rs182491843	0.000481	326	A-band	100	0.08068
103	25-29	M	HL	c.76141G>A	p.Ala25381Thr	rs763636099	0.000008	326	A-band	100	0.84345
104	25-29	M	HL	c.106827T>G	p.Ile35609Met	rs727504540	0.000337	360	M-band	100	0.53062
105	50-54	F	HL	c.88394C>T	p.Ser29465Phe	rs146181116	0.002928	331	A-band	100	0.65325
106	60-64	F	NHB	c.79612A>G	p.Thr26538Ala	rs150682764	0.000322	326	A-band	100	0.70029
107	65-69	M	HL	c.84309C>G	p.His28103Gln	rs749278779	0.000004	326	A-band	100	0.35528
108	60-64	M	NHB	c.44077C>T	p.Arg14693Cys	rs200445568	0.000169	238	I-band	100	0.68844
109	60-64	M	NHB	c.6927T>A	p.Asn2309Lys	rs147580120	0.000024	30	I-band	100	0.2335
110	60-64	M	NHB	c.81539T>C	p.Ile27180Thr	rs182126530	0.000669	326	A-band	100	0.64318
111	65-69	M	NHB	c.47737C>T	p.Leu15913Phe	rs138576504	0.000399	254	A-band	100	0.60456
112	65-69	M	NHB	c.55547T>C	p.Ile18516Thr	rs146608896	0.000467	287	A-band	100	0.78662
113	60-64	M	NHB	c.87611C>G	p.Thr29204Arg	rs72648228	0.000157	328	A-band	100	0.29647
114	60-64	M	NHB	c.2764C>T	p.Arg922Cys	rs72647862	0.000331	16	near Z-disk	99	0.54641
115	60-64	M	NHB	c.86393G>A	p.Arg28798Lys	rs781458689	0.000008	326	A-band	100	0.62838
116	60-64	M	NHB	c.62432A>G	p.Asp20811Gly	rs72646849	0.000165	304	A-band	100	0.76487
117	70-74	F	HL	c.57145G>A	p.Val19049Ile	rs750251277	0.000080	293	A-band	100	0.56694
118	70-74	F	HL	c.92444G>A	p.Cys30815Tyr	rs1185347998	0.000004	339	A-band	100	0.59375
119	80-84	M	HL	c.8509A>G	p.Ser2837Gly	rs202024134	0.000004	36	I-band	100	0.43662
120	80-84	M	HL	c.86759C>G	p.Ser28920Cys	rs1396089552	0.000004	326	A-band	100	0.36365
121	80-84	M	HL	c.92176C>T	p.Pro30726Ser	rs72648247	0.002682	339	A-band	100	0.79583
122	75-79	M	NHB	c.101245G>A	p.Val33749Met	rs201554140	0.000538	358	A-band	100	0.72631
123	75-79	M	NHB	c.4199G>C	p.Ser1400Thr	rs138506461	0.000518	24	near Z-disk	100	0.12618
124	75-79	M	NHB	c.105127C>T	p.Arg35043Cys	rs200378865	0.000462	358	A-band	100	0.79765
125	45-49	F	NHB	c.46040T>G	p.Val15347Gly	rs375367475	0.000044	248	I-band	100	0.77783
126	45-49	F	NHB	c.72782G>A	p.Arg24261Gln	rs142874389	0.000574	326	A-band	100	0.65522
127	45-49	F	NHB	c.72137C>T	p.Ala24046Val	rs146767076	0.000363	326	A-band	100	0.20419
128	65-69	F	NHB	c.97106C>T	p.Thr32369Ile	rs559194338	0.000004	348	A-band	100	0.62729
129	75-79	F	NHB	c.50390G>A	p.Arg16797His	rs200835354	0.000093	268	A-band	100	0.71084
130	65-69	F	HL	c.94851T>A	p.Asp31617Glu	rs72648256	0.002881	342	A-band	100	0.6823
131	65-69	F	HL	c.14870C>G	p.Thr4957Ser	rs72648925	0.002917	50	I-band	100	0.28911
132	65-69	M	NHB	c.101936C>G	p.Pro33979Arg	rs200238877	0.000213	358	A-band	100	0.73517
133	65-69	M	NHB	c.85691A>T	p.Lys28564Ile	rs199859344	0.000443	326	A-band	100	0.69443
134	65-69	M	NHB	c.57683G>A	p.Arg19228His	rs114711705	0.000488	295	A-band	100	0.30369

medRxiv preprint doi: https://doi.org/10.1101/2024.12.06.24318402; this version posted November 7, 2025. The copyright holder for this preprint (which was not certified by peer review) is the author/funder, who has granted medRxiv a license to display the preprint in perpetuity.
It is made available under a CC-BY 4.0 International license .

Subject ID	Age	Sex	Race-Ethnicity	Nucleotide	Amino Acid Change	dbSNP	gnomAD Allele Frequency	Exon	Band	Percent Spliced In (PSI)	REVEL Score
135	65-69	F	NHB	c.61138C>A	p.Leu20380Met	rs201167216	0.000271	304	A-band	100	0.41658
136	65-69	F	NHB	c.86911G>A	p.Gly28971Arg	rs368921501	0.000040	327	A-band	100	0.79217
137	65-69	F	NHB	c.66692G>A	p.Arg22231His	rs200971254	0.000343	316	A-band	100	0.76881
138	65-69	F	HL	c.69130C>T	p.Pro23044Ser	rs55980498	0.003619	324	A-band	100	0.83802

Supplementary Table 1: List of *TTN* missense variants. Age represents patient's age at AF diagnosis in years. M=male, F=female, HL = Hispanic/Latinx, NHB = non-Hispanic Black. Variants with a blank value in the dbSNP or gnomAD columns represent variants not present in those respective databases.

medRxiv preprint doi: https://doi.org/10.1101/2024.12.06.24318402; this version posted November 7, 2025. The copyright holder for this preprint (which was not certified by peer review) is the author/funder, who has granted medRxiv a license to display the preprint in perpetuity.
It is made available under a CC-BY 4.0 International license .

medRxiv preprint doi: https://doi.org/10.1101/2024.12.06.24318402; this version posted November 7, 2025. The copyright holder for this preprint (which was not certified by peer review) is the author/funder, who has granted medRxiv a license to display the preprint in perpetuity.
It is made available under a CC-BY 4.0 International license .

	Predicted Deleterious *TTN* Missense Absent (N=88)	Predicted Deleterious *TTN* Missense Present (N=43)	Total (N=131)	P-value
**Age at AF diagnosis (years)**	63.7 (14.5)	63.0 (12.5)	63.5 (13.8)	0.803
**Male sex**	47 (53.4%)	23 (53.5%)	70 (53.4%)	1.000
**Race/ethnicity**				0.026
Non-Hispanic Black	57 (64.8%)	36 (83.7%)	93 (71.0%)	
Hispanic/Latinx	31 (35.2%)	7 (16.3%)	38 (29.0%)	
**BMI (kg/m^2)**	33.8 (8.9)	34.9 (11.1)	34.1 (9.7)	0.540
**Diabetes**	35 (39.8%)	15 (34.9%)	50 (38.2%)	0.702
**Hypertension**	77 (87.5%)	36 (83.7%)	113 (86.3%)	0.594
**Coronary artery disease**	19 (21.6%)	13 (30.2%)	32 (24.4%)	0.288
**History of stroke/transient ischemic attack**	17 (19.3%)	9 (20.9%)	26 (19.8%)	0.819
**Congestive heart failure**	32 (36.4%)	22 (51.2%)	54 (41.2%)	0.131
**Nonischemic dilated cardiomyopathy**	6 (7.1%)	6 (15.4%)	12 (9.7%)	0.191
**Estimated glomerular filtration rate** (mg/dL)	69.1 (24.6)	68.9 (24.8)	69.1 (24.6)	0.965
**Ventricular rate**	90.9 (27.4)	103.7 (31.4)	95.3 (29.4)	0.022
**QRS interval (ms)**	97.3 (24.3)	102.7 (30.0)	99.2 (26.3)	0.292
**QTc interval (ms)**	453.9 (38.7)	470.6 (44.0)	459.6 (41.2)	0.035
**Left ventricular ejection fraction (%)**				0.144
Normal (>/=50%)	55 (62.5%)	22 (51.2%)	77 (58.8%)	
Mildly decreased (40-49%)	11 (12.5%)	3 (7.0%)	14 (10.7%)	
Moderately decreased (30-39%)	7 (8.0%)	6 (14.0%)	13 (9.9%)	
Severely decreased (20-29%)	8 (9.1%)	8 (18.6%)	16 (12.2%)	
Very severely decreased (< 20%)	7 (8.0%)	4 (9.3%)	11 (8.4%)	
**Left ventricular end diastolic diameter (mm)**	45.6 (9.2)	49.8 (8.0)	46.9 (9.0)	0.021
**Left ventricular dilatation**	9 (11.8%)	8 (22.2%)	17 (15.2%)	0.168
**Left atrial size**				0.728
Normal	26 (31.3%)	12 (28.6%)	38 (30.4%)	
Mildly dilated	22 (26.5%)	16 (38.1%)	38 (30.4%)	
Moderately dilated	22 (26.5%)	8 (19.0%)	30 (24.0%)	
Severely dilated	13 (15.7%)	6 (14.3%)	19 (15.2%)	
**Left atrial diameter (mm)**	39.8 (7.5)	41.5 (8.4)	40.4 (7.8)	0.293

Supplementary Table 2: Clinical characteristics of ethnic minority subjects with AF stratified by presence of predicted deleterious rare missense *TTN* variants. *Data are missing for the following variables: eGFR (1), electrocardiogram within 3 months of AF diagnosis (11), LVEDD (19), left atrial size (6), left atrial diameter (21). Left ventricular dilatation is defined as left ventricular end diastolic diameter greater than 2 standard deviations above the normal sex-specific mean value. Variants with a REVEL score ≥ 0.7 were defined as predicted deleterious. Continuous data are

medRxiv preprint doi: https://doi.org/10.1101/2024.12.06.24318402; this version posted November 7, 2025. The copyright holder for this preprint (which was not certified by peer review) is the author/funder, who has granted medRxiv a license to display the preprint in perpetuity.
It is made available under a CC-BY 4.0 International license .

represented as mean (standard deviation) and categorical data are represented as count (%).

Subject ID	Age	Sex	Race-Ethnicity	Nucleotide	Amino Acid Change	Exon	Band	Percent Spliced In (PSI)	REVEL Score	LVEDD (mm)	LVEF (%)
1	55-59	F	NHB	c.70250T>C	p.Ile23417Thr	326	A-band	100	0.66297	57.9	Mildly decreased (40-49%)
2	40-44	F	NHB	c.101665G>A	p.Val33889Ile	358	A-band	100	0.17295	53.2	Mildly decreased (40-49%)
3	40-44	F	NHB	c.6959G>A	p.Arg2320His	30	I-band	100	0.82403	53.2	Mildly decreased (40-49%)
4	65-69	F	NHB	c.91937A>G	p.Asn30646Ser	338	A-band	100	0.71558	56.9	Severely decreased (20-29%)
5	65-69	F	NHB	c.46693G>T	p.Ala15565Ser	250	I-band	100	0.51417	56.9	Severely decreased (20-29%)
6	55-59	M	NHB	c.44525C>T	p.Thr14842Ile	241	I-band	100	0.75347	59.0	Very severely decreased (< 20%)
7	55-59	M	NHB	c.81502C>T	p.Arg27168Cys	326	A-band	100	0.63269	59.0	Very severely decreased (< 20%)
8	40-44	F	NHB	c.69883G>A	p.Ala23295Thr	326	A-band	100	0.21907	60.3	Moderately decreased (30-39%)
9	45-49	M	NHB	c.101245G>A	p.Val33749Met	358	A-band	100	0.72631	66.5	Very severely decreased (< 20%)
10	45-49	M	NHB	c.4199G>C	p.Ser1400Thr	24	near Z-disk	100	0.12618	66.5	Very severely decreased (< 20%)
11	45-49	M	NHB	c.105127C>T	p.Arg35043Cys	358	A-band	100	0.79765	66.5	Very severely decreased (< 20%)
12	25-29	M	HL	c.64997C>T	p.Ala21666Val	311	A-band	100	0.5065	60.3	Severely decreased (20-29%)
13	25-29	M	HL	c.106349C>G	p.Thr35450Ser	358	A-band	100	0.30369	60.3	Severely decreased (20-29%)
14	25-29	M	HL	c.97760G>C	p.Arg32587Pro	350	A-band	100	0.58633	60.3	Severely decreased (20-29%)
15	25-29	M	HL	c.57165A>T	p.Glu19055Asp	293	A-band	100	0.58507	60.3	Severely decreased (20-29%)
16	25-29	M	HL	c.73168A>G	p.Thr24390Ala	326	A-band	100	0.08068	60.3	Severely decreased (20-29%)
17	25-29	M	HL	c.76141G>A	p.Ala25381Thr	326	A-band	100	0.84345	60.3	Severely decreased (20-29%)
18	25-29	M	HL	c.106827T>G	p.Ile35609Met	360	M-band	100	0.53062	60.3	Severely decreased (20-29%)
19	65-69	M	NHB	c.47737C>T	p.Leu15913Phe	254	A-band	100	0.60456	66.2	Moderately decreased (30-39%)
20	65-69	M	NHB	c.55547T>C	p.Ile18516Thr	287	A-band	100	0.78662	66.2	Moderately decreased (30-39%)

Supplementary Table 3: *TTN* missense variants in subjects meeting criteria for nonischemic dilated cardiomyopathy. Nonischemic dilated cardiomyopathy was defined by left ventricular ejection fraction <50% and left ventricular end diastolic diameter (LVEDD) greater than 2 standard deviations above the sex-specific mean, as well as coronary angiogram confirming the absence of obstructive coronary artery disease.

medRxiv preprint doi: https://doi.org/10.1101/2024.12.06.24318402; this version posted November 7, 2025. The copyright holder for this preprint (which was not certified by peer review) is the author/funder, who has granted medRxiv a license to display the preprint in perpetuity.
It is made available under a CC-BY 4.0 International license .

medRxiv preprint doi: https://doi.org/10.1101/2024.12.06.24318402; this version posted November 7, 2025. The copyright holder for this preprint (which was not certified by peer review) is the author/funder, who has granted medRxiv a license to display the preprint in perpetuity.
It is made available under a CC-BY 4.0 International license .

	Unadjusted			Partially Adjusted			Fully Adjusted		
**Characteristic**	**HR**[1]	**95% CI**[1]	**p-value**	**HR**[1]	**95% CI**[1]	**p-value**	**HR**[1]	**95% CI**[1]	**p-value**
TTN Missense Present	1.81	1.04, 3.15	0.036	1.82	1.04, 3.17	0.035	1.80	1.03, 3.15	0.039
Age (years)				0.99	0.97, 1.01	0.366	0.99	0.97, 1.01	0.430
Male sex (vs. female)				0.74	0.43, 1.27	0.268	0.69	0.38, 1.24	0.218
Race-ethnicity									
Non-Hispanic Black							—	—	
Hispanic/Latinx							1.14	0.60, 2.19	0.683
Baseline ejection fraction <50%							1.38	0.78, 2.44	0.272

[1]HR = Hazard Ratio, CI = Confidence Interval

Supplementary Table 4: Parameter estimates for univariable and multivariable Cox proportional hazard models of atrial fibrillation and heart failure-related hospitalizations. A partially adjusted multivariable model contained covariates of age and sex, and the fully adjusted model additionally accounted for race-ethnicity and ejection fraction <50% closest to AF diagnosis.

medRxiv preprint doi: https://doi.org/10.1101/2024.12.06.24318402; this version posted November 7, 2025. The copyright holder for this preprint (which was not certified by peer review) is the author/funder, who has granted medRxiv a license to display the preprint in perpetuity.
It is made available under a CC-BY 4.0 International license .

	Unadjusted			Partially Adjusted			Fully Adjusted		
**Characteristic**	**HR**[1]	**95% CI**[1]	**p-value**	**HR**[1]	**95% CI**[1]	**p-value**	**HR**[1]	**95% CI**[1]	**p-value**
TTN Missense									
None	—	—		—	—		—	—	
REVEL <0.70	1.60	0.78, 3.28	0.198	1.61	0.79, 3.26	0.188	1.60	0.80, 3.21	0.182
REVEL ≥0.70	1.92	1.04, 3.53	0.036	1.92	1.04, 3.56	0.038	1.91	1.04, 3.51	0.038
Age (years)				0.99	0.97, 1.01	0.363	0.99	0.97, 1.01	0.412
Male sex (vs. female)				0.73	0.42, 1.25	0.251	0.68	0.38, 1.23	0.204
Race-ethnicity									
Non-Hispanic Black							—	—	
Hispanic/Latinx							1.17	0.63, 2.16	0.619
Baseline ejection fraction <50%							1.35	0.77, 2.36	0.302

[1]HR = Hazard Ratio, CI = Confidence Interval

Supplementary Table 5: Cox proportional hazard models of hospitalizations related to TTN missense variants based on *in silico* prediction of impact. REVEL score of ≥0.70 indicates potentially deleterious effect. A partially adjusted multivariable model contained covariates of age and sex, and the fully adjusted model additionally accounted for race-ethnicity and ejection fraction <50% closest to AF diagnosis.

medRxiv preprint doi: https://doi.org/10.1101/2024.12.06.24318402; this version posted November 7, 2025. The copyright holder for this preprint (which was not certified by peer review) is the author/funder, who has granted medRxiv a license to display the preprint in perpetuity.
It is made available under a CC-BY 4.0 International license .

	Unadjusted			Partially Adjusted			Fully Adjusted		
**Characteristic**	**HR[1]**	**95% CI[1]**	**p-value**	**HR[1]**	**95% CI[1]**	**p-value**	**HR[1]**	**95% CI[1]**	**p-value**
TTN Missense Present	1.81	1.00, 3.29	0.051	1.84	1.01, 3.34	0.046	1.83	1.01, 3.32	0.046
Age (years)				0.99	0.97, 1.01	0.365	0.99	0.97, 1.01	0.360
Male sex (vs. female)				0.75	0.42, 1.35	0.344	0.70	0.37, 1.32	0.268
Race-ethnicity									
Non-Hispanic Black							—	—	
Hispanic/Latinx							1.17	0.59, 2.31	0.649
Baseline ejection fraction <50%							1.41	0.76, 2.61	0.280

[1]HR = Hazard Ratio, CI = Confidence Interval

Supplementary Table 6: Cox proportional hazard models of hospitalizations excluding cases with nonischemic dilated cardiomyopathy. A total of 12 subjects were excluded. A partially adjusted multivariable model contained covariates of age and sex, and the fully adjusted model additionally accounted for race-ethnicity and ejection fraction <50% closest to AF diagnosis.

medRxiv preprint doi: https://doi.org/10.1101/2024.12.06.24318402; this version posted November 7, 2025. The copyright holder for this preprint (which was not certified by peer review) is the author/funder, who has granted medRxiv a license to display the preprint in perpetuity.
It is made available under a CC-BY 4.0 International license .

Nucleotide and Protein ID	NM_001267550.2(TTN):c.98267C>T (NP_001254479.2:p.Thr32756Ile)
Allele ID	173049
Variant type	single nucleotide variant (missense)
Variant length	1 bp
Cytogenetic location	2q31.2
Genomic location	2: 178539798 (GRCh38) GRCh38 UCSC; 2: 179404525 (GRCh37) GRCh37 UCSC
Canonical SPDI	NC_000002.12:178539797:G:A
Source	https://www.ncbi.nlm.nih.gov/clinvar/variation/178164/

Supplementary Table 7: *TTN*-T32756I variant information.
