# Supplementary material for: A Titin Missense Variant Causes Atrial Fibrillation
Source: medRxiv. 2024 Dec 8:2024.12.06.24318402. Preprint. [Version 1] doi: 10.1101/2024.12.06.24318402 (PMC11643245; doi:10.1101/2024.12.06.24318402)
Supplement: Supplementary file 1 [file NIHPP2024.12.06.24318402V1-supplement-1.pdf]

medRxiv preprint doi: https://doi.org/10.1101/2024.12.06.24318402; this version posted December 8, 2024. The copyright holder for this preprint (which was not certified by peer review) is the author/funder, who has granted medRxiv a license to display the preprint in perpetuity.
It is made available under a CC-BY 4.0 International license .

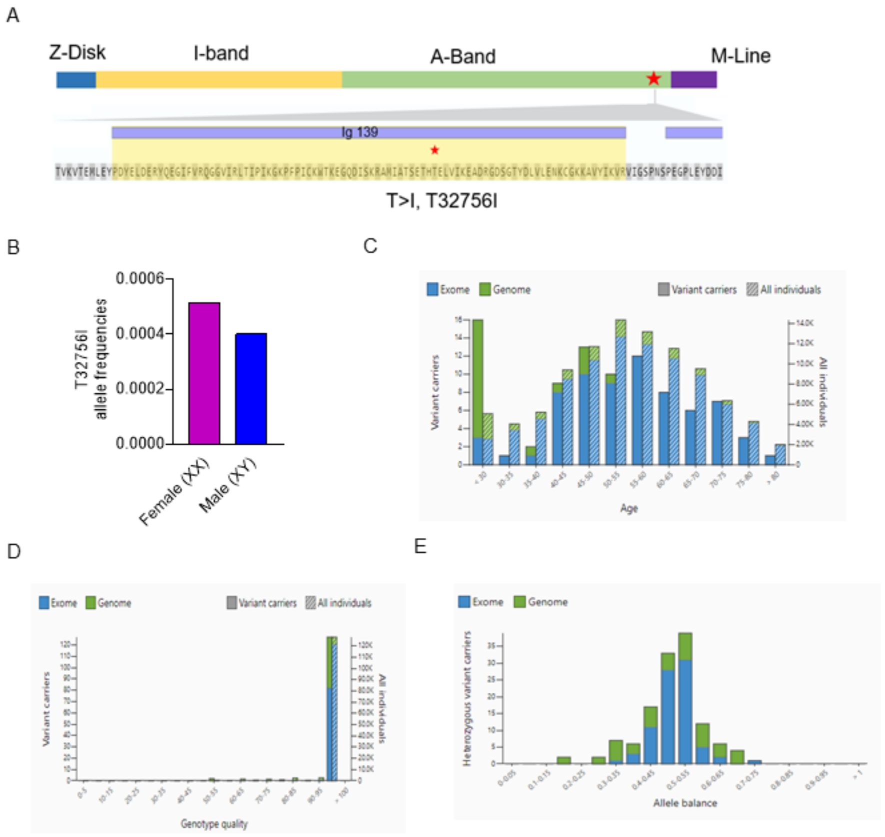

Supplementary Figure 1: *TTN*-T32756I position and distribution. (A) Location of the T23756I at Ig139 domain in the A-band of titin. (B) Allele frequencies of T3265I between sexes. (C) Age distribution of the variant carriers. (D) Genotype quality. (E) Allele balance for heterozygotes. Source: https://gnomad.broadinstitute.org/variant/2-179404525-G-A?dataset=gnomad_r2_1.

medRxiv preprint doi: https://doi.org/10.1101/2024.12.06.24318402; this version posted December 8, 2024. The copyright holder for this preprint (which was not certified by peer review) is the author/funder, who has granted medRxiv a license to display the preprint in perpetuity.
It is made available under a CC-BY 4.0 International license .

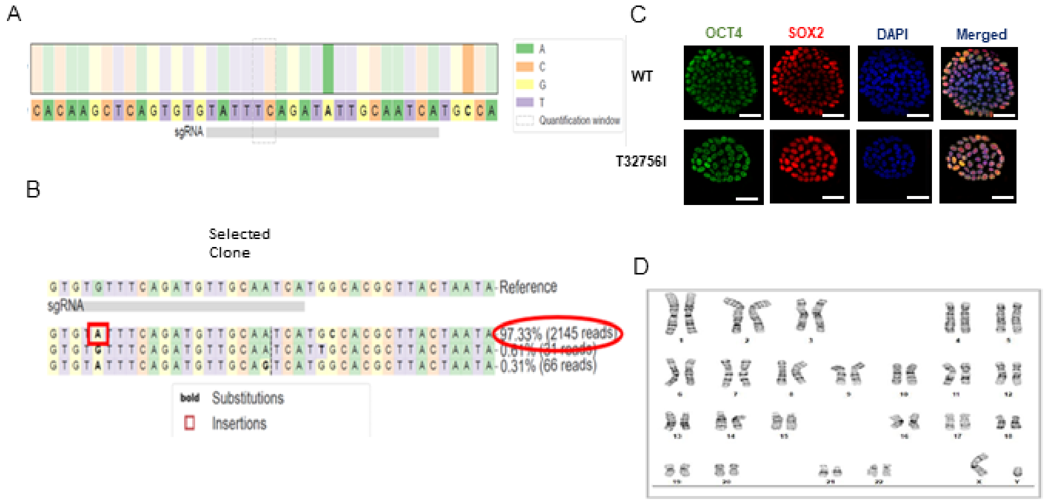

Supplementary Figure 2: Generation of iPSC-aCMs with *TTN*-T32756I. (A) *TTN* gene locus for generation of isogenic iPSCs with the T32756I variation. Guide sequence (gRNA) shown in the bottom gray box was cloned into vector to express gRNA guiding Cas9 exonuclease to the targeted protospacer adjacent motif sequence. (B) Next generation sequencing of the confirming T32756I mutation. (C) Representative immunostaining of pluripotency markers OCT4 and SOX2 in iPSCs. The 4′,6-diamidino-2-phenylindole (DAPI) indicates the nucleus. (D) Karyotype analysis of the T32756I iPSCs.

medRxiv preprint doi: https://doi.org/10.1101/2024.12.06.24318402; this version posted December 8, 2024. The copyright holder for this preprint (which was not certified by peer review) is the author/funder, who has granted medRxiv a license to display the preprint in perpetuity.
It is made available under a CC-BY 4.0 International license .

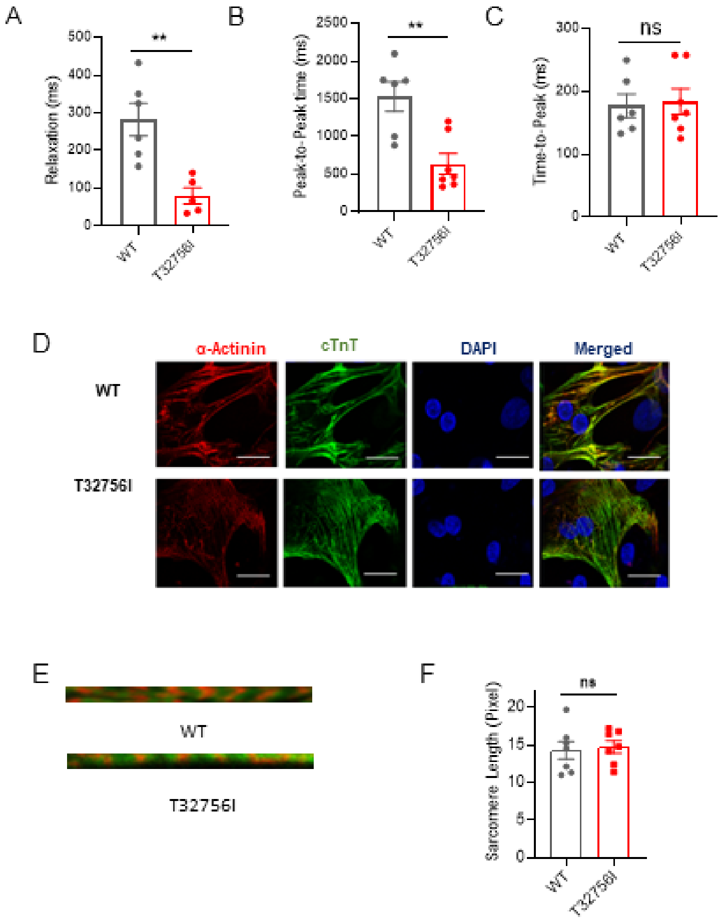

Supplementary Figure 3: Contractility and sarcomere organization of *TTN*-T32756I iPSC-aCMs. (A-C) Compared to WT, *TTN*-T32756I (Red) iPSC-aCMs show increased decreased Relaxation time (A), Contraction Amplitude (B), but no significant change to Time-to-Peak (C). (D-E) Immunostaining showing the sarcomeric organization of WT and *TTN*-T32756I iPSC-aCM by the pan-cardiomyocyte (CM) marker cardiac troponin T (cTnT; green) and α-actinin (orange). The DAPI staining indicates the nucleus. (F) Bar graph showing no change in the sarcomere length. n.s. P>0.05; **P<0.01.

medRxiv preprint doi: https://doi.org/10.1101/2024.12.06.24318402; this version posted December 8, 2024. The copyright holder for this preprint (which was not certified by peer review) is the author/funder, who has granted medRxiv a license to display the preprint in perpetuity.
It is made available under a CC-BY 4.0 International license .

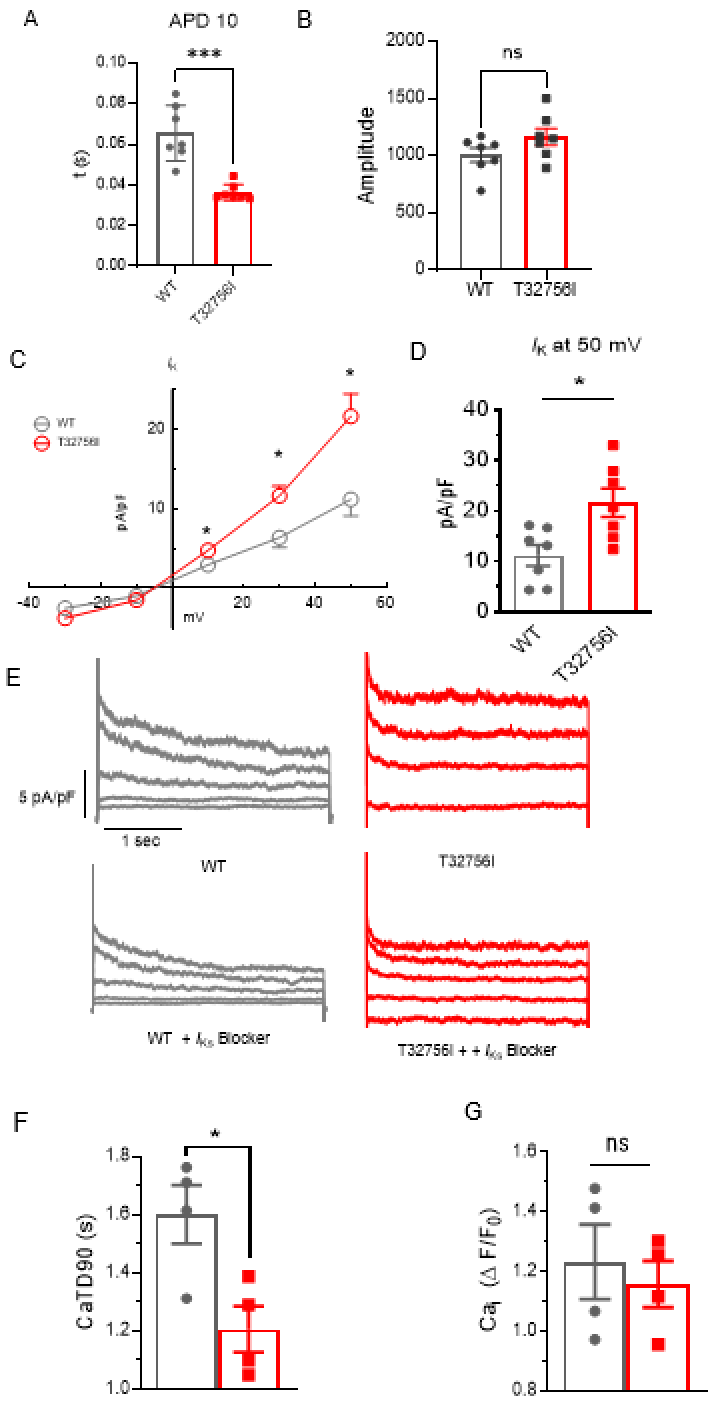

Supplementary Figure 4: *TTN*-T32756I iPSC-aCMs display anomalous action potentials, potassium currents, and calcium-handling. (A) Compared to the WT, *TTN*-T32756I shows reduction of action potential duration at the 10% (APD10) repolarization. (B) Bar graph showing no change in the amplitude of the AP. (C) Total potassium current

medRxiv preprint doi: https://doi.org/10.1101/2024.12.06.24318402; this version posted December 8, 2024. The copyright holder for this preprint (which was not certified by peer review) is the author/funder, who has granted medRxiv a license to display the preprint in perpetuity.
It is made available under a CC-BY 4.0 International license .

(I_K) and voltage relationship (I-V curves) in WT and *TTN*-T32756I iPSC-aCMs. (D) Total I_K current density at 50 mV. (E) Representative current traces at different voltages showing the isolation of the Iks current with the selective blocker HMR-1556 in both WT and *TTN*-T32756I iPSC-aCMs. (F-G) Bar graph showing that the *TTN*-T32756I iPSC-aCMs have decreased transient durations (F), but no change in the transient peak amplitudes (G) compared with the WT iPSC-aCMs. n.s. P>0.05; **P<0.05; ***P<0.001*.

medRxiv preprint doi: https://doi.org/10.1101/2024.12.06.24318402; this version posted December 8, 2024. The copyright holder for this preprint (which was not certified by peer review) is the author/funder, who has granted medRxiv a license to display the preprint in perpetuity.
It is made available under a CC-BY 4.0 International license .

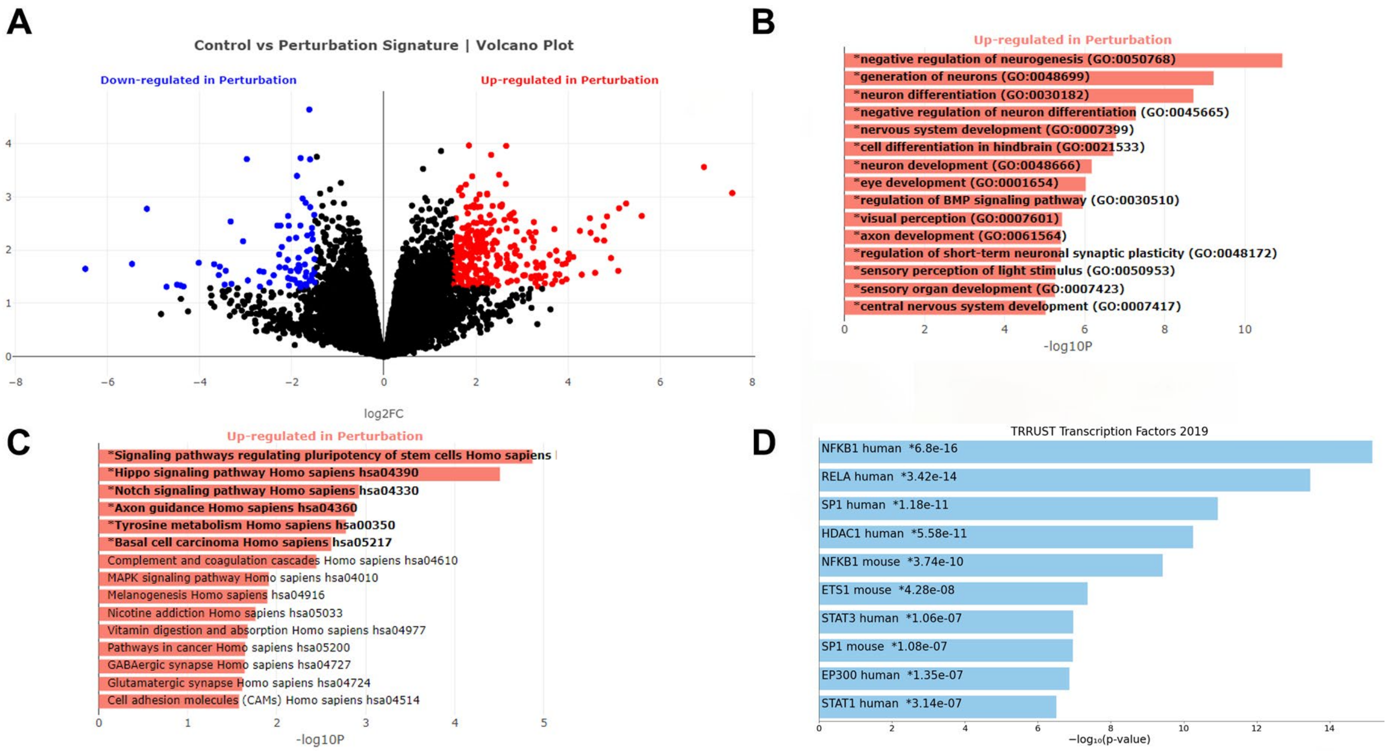

Supplementary Figure 5: Upregulated pathways in *TTN*-T32756I iPSC-aCMs with the WT. (A) Volcano plot showing spread of downregulated and upregulated differentially expressed genes (DEGs) (**B**) Top significantly enriched upregulated Gene-Ontology Biological process (GO-BP) pathways in the *TTN*-T32756I iPSC-aCMs. (**C**) Top significantly enriched upregulated Kyoto Encyclopedia of Genes and Genomes (KEGG) pathways in the *TTN*-T32756I iPSC-aCMs. (**D**) Top significantly enriched TTRUST transcription factors (TFs).

N	Age Range (yrs)	Sex	Race-Ethnicity	Nucleotide	Amino Acid Change	dbSNP	gnomAD Allele Frequency	Exon	Band	Percent Spliced In (PSI)	REVEL Score
1	56-60	F	NHB	c.70250T>C	p.Ile23417Thr	rs201836227	0.000221	326	A-band	100	0.66297
2	46-50	M	HL	c.52022G>A	p.Arg17341Gln	rs370390570	0.000116	273	A-band	100	0.60691
3	46-50	M	HL	c.62519G>A	p.Gly20840Asp	rs1326564200	0.000012	304	A-band	100	0.883
4	56-60	M	NHB	c.59248G>A	p.Gly19750Ser	rs200732032	0.000109	300	A-band	100	0.75279
5	56-60	M	NHB	c.58363G>A	p.Gly19455Ser	rs191927501	0.000157	297	A-band	100	0.80842
6	66-70	M	HL	c.57727G>C	p.Ala19243Pro	rs1313667626	0.000004	295	A-band	100	0.81369
7	41-45	F	NHB	c.101665G>A	p.Val33889Ile	rs34924609	0.003099	358	A-band	100	0.17295
8	41-45	F	NHB	c.6959G>A	p.Arg2320His	rs374615369	0.000076	30	I-band	100	0.82403
9	56-60	M	NHB	c.42145G>T	p.Val14049Leu	rs1206523368			I-band	100	0.73517
10	61-65	M	NHB	c.67808C>T	p.Ala22603Val	rs199583938	0.000036	320	A-band	100	0.42028
11	61-65	M	NHB	c.101557A>G	p.Lys33853Glu	rs727505163	0.000004	358	A-band	100	0.75074
12	76-80	F	NHB	c.93266G>A	p.Arg31089His	rs367993101	0.000028	339	A-band	100	0.87283
13	76-80	F	NHB	c.970C>T	p.Pro324Ser	rs72647845	0.000598	7	Z-disk	100	0.70603
14	56-60	M	HL	c.89426G>A	p.Arg29809Gln	rs72648238	0.000632	334	A-band	100	0.45592
15	56-60	M	HL	c.99433C>T	p.Arg33145Trp	rs1338284042	0.000004	355	A-band	100	0.61955
16	76-80	M	NHB	c.74870A>G	p.Lys24957Arg	rs760043791	0.000004	326	A-band	100	0.43483
17	76-80	M	NHB	c.70817T>C	p.Met23606Thr	rs371030086	0.000040	326	A-band	100	0.624
18	81-85	F	NHB	c.101665G>A	p.Val33889Ile	rs34924609	0.003099	358	A-band	100	0.17295
19	36-40	F	NHB	c.96605T>C	p.Val32202Ala	rs763365622	0.000004	347	A-band	100	0.54781
20	31-35	F	NHB	c.70543T>G	p.Tyr23515Asp				A-band	100	0.67962
21	61-65	M	NHB	c.4671G>A	p.Met1557Ile	rs139192633	0.000272	27	near Z-disk	100	0.26882
22	66-70	F	NHB	c.91937A>G	p.Asn30646Ser	rs72648245	0.000568	338	A-band	100	0.71558
23	66-70	F	NHB	c.46693G>T	p.Ala15565Ser	rs145520397	0.000445	250	I-band	100	0.51417
24	66-70	F	NHB	c.100396C>T	p.Arg33466Cys	rs371908649	0.000145	357	A-band	100	0.8849
25	66-70	F	NHB	c.56693G>A	p.Arg18898His	rs572453785	0.000051	291	A-band	100	0.29647
26	46-50	M	NHB	c.82061T>G	p.Val27354Gly	rs368023868	0.000036	326	A-band	100	0.77847
27	46-50	M	NHB	c.76987G>A	p.Asp25663Asn	rs143186270	0.000105	326	A-band	100	0.49396
28	81-85	F	NHB	c.8938G>A	p.Ala2980Thr	rs72647885	0.000371	38	I-band	100	0.75822
29	66-70	F	NHB	c.9077A>T	p.Asn3026Ile	rs11900987	0.000454	38	I-band	100	0.56024
30	81-85	F	HL	c.7180G>C	p.Glu2394Gln	rs537269762	0.000004	31	I-band	100	0.58254
31	46-50	M	HL	c.57586C>G	p.Leu19196Val	rs397517630	0.000171	295	A-band	100	0.50185
32	61-65	F	NHB	c.44072C>T	p.Thr14691Ile	rs1048028645			I-band	100	0.73445
33	61-65	F	NHB	c.63245C>A	p.Thr21082Asn				A-band	100	0.74377
34	66-70	F	NHB	c.101936C>G	p.Pro33979Arg	rs200238877	0.000213	358	A-band	100	0.73517
35	66-70	F	NHB	c.57683G>A	p.Arg19228His	rs114711705	0.000488	295	A-band	100	0.30369
36	66-70	F	NHB	c.85691A>T	p.Lys28564Ile	rs199859344	0.000443	326	A-band	100	0.69443
37	56-60	M	NHB	c.44525C>T	p.Thr14842Ile	rs370782364	0.000012	241	I-band	100	0.75347
38	56-60	M	NHB	c.81502C>T	p.Arg27168Cys	rs377616334	0.000028	326	A-band	100	0.63269
39	66-70	F	NHB	c.88340C>G	p.Thr29447Arg	rs140201636	0.000198	331	A-band	100	0.58883
40	66-70	F	NHB	c.87137T>G	p.Met29046Arg	rs143975327	0.000192	328	A-band	100	0.81427
41	66-70	F	NHB	c.73316C>T	p.Thr24439Ile	rs750110781	0.000008	326	A-band	100	0.32913
42	56-60	F	NHB	c.98267C>T	p.Thr32756Ile	rs199805060	0.000330	352	A-band	100	0.58758
43	61-65	M	HL	c.91573A>G	p.Ile30525Val	rs72648244	0.006222	337	A-band	100	0.18881
44	61-65	M	HL	c.72931A>G	p.Thr24311Ala	rs56201325	0.003985	326	A-band	100	0.1533

N	Age Range (yrs)	Sex	Race-Ethnicity	Nucleotide	Amino Acid Change	dbSNP	gnomAD Allele Frequency	Exon	Band	Percent Spliced In (PSI)	REVEL Score
45	61-65	F	NHB	c.8938G>A	p.Ala2980Thr	rs72647885	0.000371	38	I-band	100	0.75822
46	66-70	M	NHB	c.79612A>G	p.Thr26538Ala	rs150682764	0.000322	326	A-band	100	0.70029
47	66-70	M	HL	c.14911T>G	p.Cys4971Gly	rs537312655	0.000439	50	I-band	100	0.3358
48	56-60	M	NHB	c.43622C>T	p.Ser14541Leu	rs768180052	0.000008	236	I-band	100	0.60219
49	41-45	F	NHB	c.69883G>A	p.Ala23295Thr	rs746519147	0.000032	326	A-band	100	0.21907
50	76-80	M	NHB	c.52927C>T	p.Arg17643Trp	rs375944265	0.000060	276	A-band	100	0.75279
51	76-80	M	NHB	c.103363C>T	p.Arg34455Cys	rs72629785	0.000716	358	A-band	100	0.75687
52	76-80	M	NHB	c.81539T>C	p.Ile27180Thr	rs182126530	0.000669	326	A-band	100	0.64318
53	76-80	M	NHB	c.44077C>T	p.Arg14693Cys	rs200445568	0.000169	238	I-band	100	0.68844
54	61-65	M	NHB	c.103363C>T	p.Arg34455Cys	rs72629785	0.000716	358	A-band	100	0.75687
55	76-80	M	NHB	c.61481T>C	p.Ile20494Thr	rs374845737	0.000022	304	A-band	100	0.7293
56	51-55	F	NHB	c.54348A>T	p.Glu18116Asp	rs773746281	0.000016	281	A-band	100	0.48268
57	71-75	M	NHB	c.103906C>T	p.Arg34636Cys	rs768575577	0.000028	358	A-band	100	0.59861
58	71-75	M	NHB	c.97892A>T	p.Lys32631Ile	rs944963846	0.000004	351	A-band	100	0.56959
59	51-55	F	NHB	c.2765G>A	p.Arg922His	rs56046320	0.000703	16	near Z-disk	99	0.27141
60	51-55	F	NHB	c.60104G>A	p.Cys20035Tyr	rs774488793			A-band	100	0.71869
61	51-55	F	NHB	c.55079C>T	p.Pro18360Leu	rs192788942	0.000117	283	A-band	100	0.76881
62	91-95	M	HL	c.95414T>G	p.Phe31805Cys				A-band	100	0.73005
63	91-95	M	HL	c.74504A>G	p.Tyr24835Cys	rs201724962	0.000069	326	A-band	100	0.80901
64	56-60	M	NHB	c.78896T>A	p.Val26299Asp	rs73036377	0.000131	326	A-band	100	0.84613
65	46-50	M	NHB	c.101245G>A	p.Val33749Met	rs201554140	0.000538	358	A-band	100	0.72631
66	46-50	M	NHB	c.4199G>C	p.Ser1400Thr	rs138506461	0.000518	24	near Z-disk	100	0.12618
67	46-50	M	NHB	c.105127C>T	p.Arg35043Cys	rs200378865	0.000462	358	A-band	100	0.79765
68	51-55	M	NHB	c.2599A>G	p.Ser867Gly	rs148631577	0.000084	16	near Z-disk	99	0.27398
69	51-55	M	NHB	c.970C>T	p.Pro324Ser	rs72647845	0.000598	7	Z-disk	100	0.70603
70	56-60	F	NHB	c.69130C>T	p.Pro23044Ser	rs55980498	0.003619	324	A-band	100	0.83802
71	51-55	M	HL	c.95557C>A	p.Arg31853Ser				A-band	100	0.47777
72	51-55	M	HL	c.67989A>T	p.Leu22663Phe	rs1485610846	0.000004	320	A-band	100	0.71401
73	46-50	M	HL	c.88394C>T	p.Ser29465Phe	rs146181116	0.002928	331	A-band	100	0.65325
74	46-50	M	HL	c.89314G>A	p.Glu29772Lys	rs200503016	0.000245	334	A-band	100	0.5787
75	61-65	M	NHB	c.47737C>T	p.Leu15913Phe	rs138576504	0.000399	254	A-band	100	0.60456
76	61-65	M	NHB	c.98893G>A	p.Asp32965Asn	rs186405108	0.000044	353	A-band	100	0.68757
77	71-75	M	HL	c.93392T>G	p.Val31131Gly	rs1176407616	0.000012	339	A-band	100	0.47942
78	41-45	F	NHB	c.97760G>A	p.Arg32587His	rs55704830	0.001734	350	A-band	100	0.50341
79	56-60	F	NHB	c.95876T>A	p.Val31959Glu	rs761732372	0.000008	345	A-band	100	0.70924
80	91-95	F	HL	c.83870G>C	p.Arg27957Thr	rs148067743	0.000145	326	A-band	100	0.23913
81	51-55	F	NHB	c.61322A>G	p.Asn20441Ser	rs147580753	0.000260	304	A-band	100	0.34673
82	51-55	F	NHB	c.8938G>A	p.Ala2980Thr	rs72647885	0.000371	38	I-band	100	0.75822
83	66-70	F	HL	c.102030T>G	p.Ser34010Arg	rs1296387134	0.000024	358	A-band	100	0.69273
84	61-65	F	HL	c.102427A>T	p.Met34143Leu	rs371226574	0.000004	358	A-band	100	0.46781
85	61-65	F	HL	c.96928A>C	p.Thr32310Pro	rs542208825	0.000024	348	A-band	100	0.46444
86	61-65	F	HL	c.56315C>T	p.Thr18772Ile	rs370118111	0.000008	289	A-band	100	0.57482
87	61-65	M	NHB	c.9077A>T	p.Asn3026Ile	rs11900987	0.000454	38	I-band	100	0.56024
88	61-65	M	NHB	c.82411G>A	p.Gly27471Ser	rs757130634			A-band	100	0.31778
89	71-75	M	NHB	c.98893G>C	p.Asp32965His	rs186405108	0.000213	353	A-band	100	0.70276

N	Age Range (yrs)	Sex	Race-Ethnicity	Nucleotide	Amino Acid Change	dbSNP	gnomAD Allele Frequency	Exon	Band	Percent Spliced In (PSI)	REVEL Score
90	71-75	M	NHB	c.72137C>T	p.Ala24046Val	rs146767076	0.000363	326	A-band	100	0.20419
91	71-75	M	NHB	c.72782G>A	p.Arg24261Gln	rs142874389	0.000574	326	A-band	100	0.65522
92	71-75	M	NHB	c.55951G>A	p.Glu18651Lys				A-band	100	0.6251
93	71-75	M	NHB	c.44965A>G	p.Ile14989Val	rs755040094	0.000004	244	I-band	100	0.50496
94	71-75	M	NHB	c.88973T>C	p.Ile29658Thr	rs750026544	0.000024	333	A-band	100	0.16972
95	61-65	M	NHB	c.106439A>G	p.His35480Arg	rs766337455			M-band	100	0.25558
96	56-60	F	NHB	c.69383C>A	p.Ser23128Tyr	rs72646882	0.000576	324	A-band	100	0.76554
97	71-75	F	NHB	c.47770T>A	p.Leu15924Met				A-band	100	0.83636
98	26-30	M	HL	c.64997C>T	p.Ala21666Val	rs1396380194	0.000004	311	A-band	100	0.5065
99	26-30	M	HL	c.106349C>G	p.Thr35450Ser	rs371022420	0.000045	358	A-band	100	0.30369
100	26-30	M	HL	c.97760G>C	p.Arg32587Pro	rs55704830	0.000393	350	A-band	100	0.58633
101	26-30	M	HL	c.57165A>T	p.Glu19055Asp	rs1263660973	0.000004	293	A-band	100	0.58507
102	26-30	M	HL	c.73168A>G	p.Thr24390Ala	rs182491843	0.000481	326	A-band	100	0.08068
103	26-30	M	HL	c.76141G>A	p.Ala25381Thr	rs763636099	0.000008	326	A-band	100	0.84345
104	26-30	M	HL	c.106827T>G	p.Ile35609Met	rs727504540	0.000337	360	M-band	100	0.53062
105	51-55	F	HL	c.88394C>T	p.Ser29465Phe	rs146181116	0.002928	331	A-band	100	0.65325
106	61-65	F	NHB	c.79612A>G	p.Thr26538Ala	rs150682764	0.000322	326	A-band	100	0.70029
107	66-70	M	HL	c.84309C>G	p.His28103Gln	rs749278779	0.000004	326	A-band	100	0.35528
108	61-65	M	NHB	c.44077C>T	p.Arg14693Cys	rs200445568	0.000169	238	I-band	100	0.68844
109	61-65	M	NHB	c.6927T>A	p.Asn2309Lys	rs147580120	0.000024	30	I-band	100	0.2335
110	61-65	M	NHB	c.81539T>C	p.Ile27180Thr	rs182126530	0.000669	326	A-band	100	0.64318
111	66-70	M	NHB	c.47737C>T	p.Leu15913Phe	rs138576504	0.000399	254	A-band	100	0.60456
112	66-70	M	NHB	c.55547T>C	p.Ile18516Thr	rs146608896	0.000467	287	A-band	100	0.78662
113	61-65	M	NHB	c.87611C>G	p.Thr29204Arg	rs72648228	0.000157	328	A-band	100	0.29647
114	61-65	M	NHB	c.2764C>T	p.Arg922Cys	rs72647862	0.000331	16	near Z-disk	99	0.54641
115	61-65	M	NHB	c.86393G>A	p.Arg28798Lys	rs781458689	0.000008	326	A-band	100	0.62838
116	61-65	M	NHB	c.62432A>G	p.Asp20811Gly	rs72646849	0.000165	304	A-band	100	0.76487
117	71-75	F	HL	c.57145G>A	p.Val19049Ile	rs750251277	0.000080	293	A-band	100	0.56694
118	71-75	F	HL	c.92444G>A	p.Cys30815Tyr	rs1185347998	0.000004	339	A-band	100	0.59375
119	81-85	M	HL	c.8509A>G	p.Ser2837Gly	rs202024134	0.000004	36	I-band	100	0.43662
120	81-85	M	HL	c.86759C>G	p.Ser28920Cys	rs1396089552	0.000004	326	A-band	100	0.36365
121	81-85	M	HL	c.92176C>T	p.Pro30726Ser	rs72648247	0.002682	339	A-band	100	0.79583
122	76-80	M	NHB	c.101245G>A	p.Val33749Met	rs201554140	0.000538	358	A-band	100	0.72631
123	76-80	M	NHB	c.4199G>C	p.Ser1400Thr	rs138506461	0.000518	24	near Z-disk	100	0.12618
124	76-80	M	NHB	c.105127C>T	p.Arg35043Cys	rs200378865	0.000462	358	A-band	100	0.79765
125	46-50	F	NHB	c.46040T>G	p.Val15347Gly	rs375367475	0.000044	248	I-band	100	0.77783
126	46-50	F	NHB	c.72782G>A	p.Arg24261Gln	rs142874389	0.000574	326	A-band	100	0.65522
127	46-50	F	NHB	c.72137C>T	p.Ala24046Val	rs146767076	0.000363	326	A-band	100	0.20419
128	66-70	F	NHB	c.97106C>T	p.Thr32369Ile	rs559194338	0.000004	348	A-band	100	0.62729
129	76-80	F	NHB	c.50390G>A	p.Arg16797His	rs200835354	0.000093	268	A-band	100	0.71084
130	66-70	F	HL	c.94851T>A	p.Asp31617Glu	rs72648256	0.002881	342	A-band	100	0.6823
131	66-70	F	HL	c.14870C>G	p.Thr4957Ser	rs72648925	0.002917	50	I-band	100	0.28911
132	66-70	M	NHB	c.101936C>G	p.Pro33979Arg	rs200238877	0.000213	358	A-band	100	0.73517
133	66-70	M	NHB	c.85691A>T	p.Lys28564Ile	rs199859344	0.000443	326	A-band	100	0.69443
134	66-70	M	NHB	c.57683G>A	p.Arg19228His	rs114711705	0.000488	295	A-band	100	0.30369

N	Age Range (yrs)	Sex	Race-Ethnicity	Nucleotide	Amino Acid Change	dbSNP	gnomAD Allele Frequency	Exon	Band	Percent Spliced In (PSI)	REVEL Score
135	61-65	F	NHB	c.61138C>A	p.Leu20380Met	rs201167216	0.000271	304	A-band	100	0.41658
136	61-65	F	NHB	c.86911G>A	p.Gly28971Arg	rs368921501	0.000040	327	A-band	100	0.79217
137	61-65	F	NHB	c.66692G>A	p.Arg22231His	rs200971254	0.000343	316	A-band	100	0.76881
138	61-65	F	HL	c.69130C>T	p.Pro23044Ser	rs55980498	0.003619	324	A-band	100	0.83802

Supplementary Table 1: List of *TTN* missense variants. Age range represents patient's approximate age at AF diagnosis in years. M=male, F=female, HL = Hispanic/Latinx, NHB = non-Hispanic Black. Variants with a blank value in the dbSNP or gnomAD columns represent variants not present in those respective databases.

medRxiv preprint doi: https://doi.org/10.1101/2024.12.06.24318402; this version posted December 8, 2024. The copyright holder for this preprint (which was not certified by peer review) is the author/funder, who has granted medRxiv a license to display the preprint in perpetuity.
It is made available under a CC-BY 4.0 International license .

	Predicted Deleterious *TTN* Missense Absent (N=88)	Predicted Deleterious *TTN* Missense Present (N=43)	Total (N=131)	P-value
**Mean age at AF diagnosis (years)**	63.7 (14.5)	63.0 (12.5)	63.5 (13.8)	0.803
**Male sex**	47 (53.4%)	23 (53.5%)	70 (53.4%)	1.000
**Race/ethnicity**				0.026
Non-Hispanic Black	57 (64.8%)	36 (83.7%)	93 (71.0%)	
Hispanic/Latinx	31 (35.2%)	7 (16.3%)	38 (29.0%)	
**BMI (kg/m^2)**	33.8 (8.9)	34.9 (11.1)	34.1 (9.7)	0.540
**Diabetes**	35 (39.8%)	15 (34.9%)	50 (38.2%)	0.702
**Hypertension**	77 (87.5%)	36 (83.7%)	113 (86.3%)	0.594
**Coronary artery disease**	19 (21.6%)	13 (30.2%)	32 (24.4%)	0.288
**History of stroke/transient ischemic attack**	17 (19.3%)	9 (20.9%)	26 (19.8%)	0.819
**Congestive heart failure**	32 (36.4%)	22 (51.2%)	54 (41.2%)	0.131
**Nonischemic dilated cardiomyopathy**	6 (7.1%)	6 (15.4%)	12 (9.7%)	0.191
**Estimated glomerular filtration rate** (mg/dL)	69.1 (24.6)	68.9 (24.8)	69.1 (24.6)	0.965
**Ventricular rate**	90.9 (27.4)	103.7 (31.4)	95.3 (29.4)	0.022
**QRS interval (ms)**	97.3 (24.3)	102.7 (30.0)	99.2 (26.3)	0.292
**QTc interval (ms)**	453.9 (38.7)	470.6 (44.0)	459.6 (41.2)	0.035
**Left ventricular ejection fraction (%)**				0.144
Normal (>/=50%)	55 (62.5%)	22 (51.2%)	77 (58.8%)	
Mildly decreased (40-49%)	11 (12.5%)	3 (7.0%)	14 (10.7%)	
Moderately decreased (30-39%)	7 (8.0%)	6 (14.0%)	13 (9.9%)	
Severely decreased (20-29%)	8 (9.1%)	8 (18.6%)	16 (12.2%)	
Very severely decreased (< 20%)	7 (8.0%)	4 (9.3%)	11 (8.4%)	
**Left ventricular end diastolic diameter (mm)**	45.6 (9.2)	49.8 (8.0)	46.9 (9.0)	0.021
**Left ventricular dilatation**	9 (11.8%)	8 (22.2%)	17 (15.2%)	0.168
**Left atrial size**				0.728
Normal	26 (31.3%)	12 (28.6%)	38 (30.4%)	
Mildly dilated	22 (26.5%)	16 (38.1%)	38 (30.4%)	
Moderately dilated	22 (26.5%)	8 (19.0%)	30 (24.0%)	
Severely dilated	13 (15.7%)	6 (14.3%)	19 (15.2%)	
**Left atrial diameter (mm)**	39.8 (7.5)	41.5 (8.4)	40.4 (7.8)	0.293

Supplementary Table 2: Clinical characteristics of ethnic minority subjects with AF stratified by presence of predicted deleterious rare missense *TTN* variants. *Data are missing for the following variables: eGFR (1), electrocardiogram within 3 months of AF diagnosis (11), LVEDD (19), left atrial size (6), left atrial diameter (21). Left ventricular dilatation is defined as left ventricular end diastolic diameter greater than 2 standard deviations above the normal sex-specific mean value. Variants with a REVEL score ≥ 0.7

medRxiv preprint doi: https://doi.org/10.1101/2024.12.06.24318402; this version posted December 8, 2024. The copyright holder for this preprint (which was not certified by peer review) is the author/funder, who has granted medRxiv a license to display the preprint in perpetuity.
It is made available under a CC-BY 4.0 International license .

were defined as predicted deleterious. Continuous data are represented as mean (standard deviation) and categorical data are represented as count (%).

N	Age Range (yrs)	Sex	Race-Ethnicity	Nucleotide	Amino Acid Change	Exon	Band	Percent Spliced In (PSI)	REVEL Score	LVEDD (mm)	LVEF (%)
1	56-60	F	NHB	c.70250T>C	p.Ile23417Thr	326	A-band	100	0.66297	57.9	Mildly decreased (40-49%)
2	41-45	F	NHB	c.101665G>A	p.Val33889Ile	358	A-band	100	0.17295	53.2	Mildly decreased (40-49%)
3	41-45	F	NHB	c.6959G>A	p.Arg2320His	30	I-band	100	0.82403	53.2	Mildly decreased (40-49%)
4	66-70	F	NHB	c.91937A>G	p.Asn30646Ser	338	A-band	100	0.71558	56.9	Severely decreased (20-29%)
5	66-70	F	NHB	c.46693G>T	p.Ala15565Ser	250	I-band	100	0.51417	56.9	Severely decreased (20-29%)
6	56-60	M	NHB	c.44525C>T	p.Thr14842Ile	241	I-band	100	0.75347	59.0	Very severely decreased (< 20%)
7	56-60	M	NHB	c.81502C>T	p.Arg27168Cys	326	A-band	100	0.63269	59.0	Very severely decreased (< 20%)
8	41-45	F	NHB	c.69883G>A	p.Ala23295Thr	326	A-band	100	0.21907	60.3	Moderately decreased (30-39%)
9	46-50	M	NHB	c.101245G>A	p.Val33749Met	358	A-band	100	0.72631	66.5	Very severely decreased (< 20%)
10	46-50	M	NHB	c.4199G>C	p.Ser1400Thr	24	near Z-disk	100	0.12618	66.5	Very severely decreased (< 20%)
11	46-50	M	NHB	c.105127C>T	p.Arg35043Cys	358	A-band	100	0.79765	66.5	Very severely decreased (< 20%)
12	26-30	M	HL	c.64997C>T	p.Ala21666Val	311	A-band	100	0.5065	60.3	Severely decreased (20-29%)
13	26-30	M	HL	c.106349C>G	p.Thr35450Ser	358	A-band	100	0.30369	60.3	Severely decreased (20-29%)
14	26-30	M	HL	c.97760G>C	p.Arg32587Pro	350	A-band	100	0.58633	60.3	Severely decreased (20-29%)
15	26-30	M	HL	c.57165A>T	p.Glu19055Asp	293	A-band	100	0.58507	60.3	Severely decreased (20-29%)
16	26-30	M	HL	c.73168A>G	p.Thr24390Ala	326	A-band	100	0.08068	60.3	Severely decreased (20-29%)
17	26-30	M	HL	c.76141G>A	p.Ala25381Thr	326	A-band	100	0.84345	60.3	Severely decreased (20-29%)
18	26-30	M	HL	c.106827T>G	p.Ile35609Met	360	M-band	100	0.53062	60.3	Severely decreased (20-29%)
19	66-70	M	NHB	c.47737C>T	p.Leu15913Phe	254	A-band	100	0.60456	66.2	Moderately decreased (30-39%)
20	66-70	M	NHB	c.55547T>C	p.Ile18516Thr	287	A-band	100	0.78662	66.2	Moderately decreased (30-39%)

Supplementary Table 3: *TTN* missense variants in subjects meeting criteria for nonischemic dilated cardiomyopathy. Nonischemic dilated cardiomyopathy was defined by left ventricular ejection fraction <50% and left ventricular end diastolic diameter (LVEDD) greater than 2 standard deviations above the sex-specific mean, as well as coronary angiogram confirming the absence of obstructive coronary artery disease.

medRxiv preprint doi: https://doi.org/10.1101/2024.12.06.24318402; this version posted December 8, 2024. The copyright holder for this preprint
(which was not certified by peer review) is the author/funder, who has granted medRxiv a license to display the preprint in perpetuity.
It is made available under a CC-BY 4.0 International license .

	Unadjusted			Partially Adjusted			Fully Adjusted		
**Characteristic**	**HR**[1]	**95% CI**[1]	**p-value**	**HR**[1]	**95% CI**[1]	**p-value**	**HR**[1]	**95% CI**[1]	**p-value**
TTN Missense Present	1.81	1.04, 3.15	0.036	1.82	1.04, 3.17	0.035	1.80	1.03, 3.15	0.039
Age (years)				0.99	0.97, 1.01	0.366	0.99	0.97, 1.01	0.430
Male sex (vs. female)				0.74	0.43, 1.27	0.268	0.69	0.38, 1.24	0.218
Race-ethnicity									
Non-Hispanic Black							—	—	
Hispanic/Latinx							1.14	0.60, 2.19	0.683
Baseline ejection fraction <50%							1.38	0.78, 2.44	0.272

[1]HR = Hazard Ratio, CI = Confidence Interval

Supplementary Table 4: Parameter estimates for univariable and multivariable Cox proportional hazard models of atrial fibrillation and heart failure-related hospitalizations. A partially adjusted multivariable model contained covariates of age and sex, and the fully adjusted model additionally accounted for race-ethnicity and ejection fraction <50% closest to AF diagnosis.

medRxiv preprint doi: https://doi.org/10.1101/2024.12.06.24318402; this version posted December 8, 2024. The copyright holder for this preprint (which was not certified by peer review) is the author/funder, who has granted medRxiv a license to display the preprint in perpetuity.
It is made available under a CC-BY 4.0 International license .

	Unadjusted			Partially Adjusted			Fully Adjusted		
**Characteristic**	**HR**[1]	**95% CI**[1]	**p-value**	**HR**[1]	**95% CI**[1]	**p-value**	**HR**[1]	**95% CI**[1]	**p-value**
TTN Missense									
None	—	—		—	—		—	—	
REVEL <0.70	1.60	0.78, 3.28	0.198	1.61	0.79, 3.26	0.188	1.60	0.80, 3.21	0.182
REVEL ≥0.70	1.92	1.04, 3.53	0.036	1.92	1.04, 3.56	0.038	1.91	1.04, 3.51	0.038
Age (years)				0.99	0.97, 1.01	0.363	0.99	0.97, 1.01	0.412
Male sex (vs. female)				0.73	0.42, 1.25	0.251	0.68	0.38, 1.23	0.204
Race-ethnicity									
Non-Hispanic Black							—	—	
Hispanic/Latinx							1.17	0.63, 2.16	0.619
Baseline ejection fraction <50%							1.35	0.77, 2.36	0.302

[1]HR = Hazard Ratio, CI = Confidence Interval

Supplementary Table 5: Cox proportional hazard models of hospitalizations related to TTN missense variant based on *in silico* prediction of impact. REVEL score of ≥0.70 indicates potentially deleterious effect. A partially adjusted multivariable model contained covariates of age and sex, and the fully adjusted model additionally accounted for race-ethnicity and ejection fraction <50% closest to AF diagnosis.

medRxiv preprint doi: https://doi.org/10.1101/2024.12.06.24318402; this version posted December 8, 2024. The copyright holder for this preprint (which was not certified by peer review) is the author/funder, who has granted medRxiv a license to display the preprint in perpetuity.
It is made available under a CC-BY 4.0 International license .

	Unadjusted			Partially Adjusted			Fully Adjusted		
**Characteristic**	**HR**[1]	**95% CI**[1]	**p-value**	**HR**[1]	**95% CI**[1]	**p-value**	**HR**[1]	**95% CI**[1]	**p-value**
TTN Missense Present	1.81	1.00, 3.29	0.051	1.84	1.01, 3.34	0.046	1.83	1.01, 3.32	0.046
Age (years)				0.99	0.97, 1.01	0.365	0.99	0.97, 1.01	0.360
Male sex (vs. female)				0.75	0.42, 1.35	0.344	0.70	0.37, 1.32	0.268
Race-ethnicity									
Non-Hispanic Black							—	—	
Hispanic/Latinx							1.17	0.59, 2.31	0.649
Baseline ejection fraction <50%							1.41	0.76, 2.61	0.280

[1]HR = Hazard Ratio, CI = Confidence Interval

Supplementary Table 6: Cox proportional hazard models of hospitalizations excluding cases with nonischemic dilated cardiomyopathy. A total of 12 subjects were excluded. A partially adjusted multivariable model contained covariates of age and sex, and the fully adjusted model additionally accounted for race-ethnicity and ejection fraction <50% closest to AF diagnosis.

medRxiv preprint doi: https://doi.org/10.1101/2024.12.06.24318402; this version posted December 8, 2024. The copyright holder for this preprint (which was not certified by peer review) is the author/funder, who has granted medRxiv a license to display the preprint in perpetuity.
It is made available under a CC-BY 4.0 International license .

Nucleotide and Protein ID	NM_001267550.2(TTN):c.98267C>T (NP_001254479.2:p.Thr32756Ile)
Allele ID	173049
Variant type	single nucleotide variant (missense)
Variant length	1 bp
Cytogenetic location	2q31.2
Genomic location	2: 178539798 (GRCh38) GRCh38 UCSC; 2: 179404525 (GRCh37) GRCh37 UCSC
Canonical SPDI	NC_000002.12:178539797:G:A
Source	https://www.ncbi.nlm.nih.gov/clinvar/variation/178164/

Supplementary Table 7: *TTN*-T32756I variant information.
